# The effect of psychiatric decision unit services on inpatient admissions and mental health presentations in emergency departments: an interrupted time series analysis from two cities and one rural area in England

**DOI:** 10.1017/S2045796024000209

**Published:** 2024-03-21

**Authors:** J. G. Smith, K. Anderson, G. Clarke, C. Crowe, L. P. Goldsmith, H. Jarman, S. Johnson, J. Lomani, D. McDaid, A. Park, K. Turner, S. Gillard

**Affiliations:** 1 Population Health Research Institute, St George’s, University of London, London, UK; 2 Clinical Research Unit, South West London & St George’s Mental Health Trust, Springfield University Hospital, London, UK; 3 Department of Psychology, Middlesex University, London, UK; 4 Improvement Analytics Unit, The Health Foundation, London, UK; 5 Sunflowers Court Inpatient Unit, North East London NHS Foundation Trust, Goodmayes Hospital, Ilford, UK; 6 Emergency Department, St George’s University Hospitals NHS Foundation Trust, London, UK; 7 NIHR Mental Health Policy Research Unit, Division of Psychiatry, University College London, London, UK; 8 Early Intervention Service, Camden and Islington NHS Foundation Trust, London, UK; 9 NHS England and NHS Improvement, London, UK; 10 Care Policy and Evaluation Centre, Department of Health Policy, London School of Economics and Political Science, London, UK; 11 School of Health and Psychological Sciences, City, University of London, London, UK

**Keywords:** emergency departments, emergency psychiatry, health service research, inpatient psychiatry, psychiatric services

## Abstract

**Aims:**

High-quality evidence is lacking for the impact on healthcare utilisation of short-stay alternatives to psychiatric inpatient services for people experiencing acute and/or complex mental health crises (known in England as psychiatric decision units [PDUs]). We assessed the extent to which changes in psychiatric hospital and emergency department (ED) activity were explained by implementation of PDUs in England using a quasi-experimental approach.

**Methods:**

We conducted an interrupted time series (ITS) analysis of weekly aggregated data pre- and post-PDU implementation in one rural and two urban sites using segmented regression, adjusting for temporal and seasonal trends. Primary outcomes were changes in the number of voluntary inpatient admissions to (acute) adult psychiatric wards and number of ED adult mental health-related attendances in the 24 months post-PDU implementation compared to that in the 24 months pre-PDU implementation.

**Results:**

The two PDUs (one urban and one rural) with longer (average) stays and high staff-to-patient ratios observed post-PDU decreases in the pattern of weekly voluntary psychiatric admissions relative to pre-PDU trend (Rural: −0.45%/week, 95% confidence interval [CI] = −0.78%, −0.12%; Urban: −0.49%/week, 95% CI = −0.73%, −0.25%); PDU implementation in each was associated with an estimated 35–38% reduction in total voluntary admissions in the post-PDU period. The (urban) PDU with the highest throughput, lowest staff-to-patient ratio and shortest average stay observed a 20% (−20.4%, CI = −29.7%, −10.0%) level reduction in mental health-related ED attendances post-PDU, although there was little impact on long-term trend. Pooled analyses across sites indicated a significant reduction in the number of voluntary admissions following PDU implementation (−16.6%, 95% CI = −23.9%, −8.5%) but no significant (long-term) trend change (−0.20%/week, 95% CI = −0.74%, 0.34%) and no short- (−2.8%, 95% CI = −19.3%, 17.0%) or long-term (0.08%/week, 95% CI = −0.13, 0.28%) effects on mental health-related ED attendances. Findings were largely unchanged in secondary (ITS) analyses that considered the introduction of other service initiatives in the study period.

**Conclusions:**

The introduction of PDUs was associated with an immediate reduction of voluntary psychiatric inpatient admissions. The extent to which PDUs change long-term trends of voluntary psychiatric admissions or impact on psychiatric presentations at ED may be linked to their configuration. PDUs with a large capacity, short length of stay and low staff-to-patient ratio can positively impact ED mental health presentations, while PDUs with longer length of stay and higher staff-to-patient ratios have potential to reduce voluntary psychiatric admissions over an extended period. Taken as a whole, our analyses suggest that when establishing a PDU, consideration of the primary crisis-care need that underlies the creation of the unit is key.

## Introduction

In many countries, much of the funding allocated to mental health service provision is expended on acute psychiatric care (World Health Organization, 2022). Financial and operational pressures on mental health crisis-care services internationally continue to grow, however, particularly with respect to inpatient hospital care (Tyrer *et al.*, 2017; Wyatt *et al.*, 2019) and (increasing) mental health-related attendances at emergency departments (EDs) (Baracaia *et al.*, 2020; Santillanes *et al.*, 2020; Tran *et al.*, 2020). Bed occupancy in National Health Service (NHS) psychiatric inpatient facilities in England is around 90%, above recommended levels (85%), with 9 out of 10 wards operating above the recommended occupancy rate and expensive out-of-area placements on the rise (Crisp *et al.*, 2016; Wyatt *et al.*, 2019). Psychiatric presentations at English EDs have increased markedly in recent years, more than doubling from 2010 to 2020 (NHS England, 2021). Compared with those presenting with other complaints, individuals seeking emergency psychiatric care are more likely to arrive via ambulance or police referral (Baracaia *et al.*, 2020), to wait longer or to leave the ED without being seen at all (Beeknoo and Jones, 2016; Ross *et al.*, 2019). Further, frequent ED attendance is associated with previous contact with specialist mental health services and with previous admission to an acute hospital for a mental health condition (Care Quality Commission, 2015), with repeat attenders at greater risk of psychiatric inpatient admissions (Okorie *et al.*, 2011).

In recent years, several hospital- and community-based developments in emergency care have been implemented across various countries to support people with urgent mental health needs, including psychiatric liaison services, mental health triage wards (inpatient facilities with a capped length of stay) and crisis resolution and home treatment (CRHT). These have had some success in decreasing waiting times and unplanned departures from ED (Evans *et al.*, 2019) and, more broadly, reducing psychiatric inpatient service use and care costs (Johnson *et al.*, 2022). But psychiatric liaison service provision remains heterogeneous across the system, and at an individual service provider level, often inconsistent with the size and composition of the acute hospital it serves (Wand *et al.*, 2016). Further, the introduction of mental health triage wards does not always significantly improve readmission rates and average lengths of inpatient stay compared with standard models of care (Da Costa *et al.*, 2021), while CRHT teams are not well suited to help individuals at very high risk to themselves and/or others (Johnson *et al.*, 2022).

To help address shortcomings in emergency mental healthcare, a range of psychiatric emergency service (PES) approaches have been developed and described internationally, whereby additional (specialist) crisis care offering individuals extended assessment and diversion following ED attendance is provided (usually within a 24-hour period), with the objective of stabilising a crisis and mitigating the risk of inpatient admission (Braitberg *et al.*, 2018; Johnson *et al.*, 2022). More recently, a number of psychiatric decision units (PDUs) have emerged in England as interim short-stay alternatives to psychiatric inpatient services for people experiencing acute and/or complex mental health crises (Goldsmith *et al.*, 2021). These hospital-based, 24-hour, non-bedded units are typically small, with between four- and eight-person capacity, and offer a stay of 12–72 hours. PDUs are staffed by senior mental health nurses and healthcare assistants, with input from psychiatrists, and have a high staff-to-patient ratio (ranging from 1:1 to 1:4) (Goldsmith *et al.*, 2021). These units are accessible to adults aged ≥18 years experiencing complex psychiatric crises who present an immediate safety risk to themselves or others and might otherwise be admitted into a psychiatric acute inpatient bed. Referral to a PDU typically follows an initial assessment by psychiatric liaison service teams in ED or via CRHT teams or street triage services (i.e., outreach services run by mental health professionals and police which provide expertise through an in-person mobile unit (Kirubarajan *et al.*, 2018)).

PDUs aim to provide service users enhanced mental health assessments, short-term support and signposting/referral to onward services in a calm and safe environment and, thus, relieve pressure on (and waiting times in) emergency services by diverting service users with psychiatric presentations from ED (when appropriate) and reducing reliance on admissions to acute psychiatric inpatient care, particularly short-stay, voluntary admissions (Goldsmith *et al.*, 2021; Trethewey *et al.*, 2019). The direct working relationship of PDUs with CRHT teams and street triage services offers a crisis care pathway that can help prevent avoidable mental health-related ED attendances. Further, the provision of a prolonged, more informed assessment of needs and risk – particularly for service users presenting with high psychiatric risk who are disproportionately represented in repeat attenders at ED (Beck *et al.*, 2016; Fernandes, 2011) – better enables referral to appropriate community and/or specialist services early in a mental health crisis (Trethewey *et al.*, 2019). This is intended to break the cycle of repeat presentations in ED, and thus, has the potential to reduce overall mental-health related attendance rates, the number of psychiatric liaison episodes stemming from ED attendance and the use of other ED-referring services for mental health-related attendances, such as police and ambulance services.

A recent systematic review examining the effectiveness of short-stay mental health crisis units suggested they potentially achieve their primary goals of reducing ED waiting times and decreasing psychiatric inpatient admissions, although marked differences in the operational structure of the examined crisis units complicated comparisons (Anderson *et al.*, 2022). However, studies of short-stay crisis units have typically been observational in design, reporting simple pre- and post-PDU comparisons of relevant activity, and it is unclear if units influence outcomes beyond underlying secular trends. The aim of the present study was to strengthen the current evidence base regarding short-stay mental health crisis units by identifying the impact of PDUs on mental health crisis-care pathways in England using an interrupted time series (ITS) approach. We used this quasi-experimental method to examine the extent to which changes in service activity relating to (voluntary) psychiatric admissions and ED psychiatric presentations were explained by PDU implementation at three different sites (two urban and one rural).

## Methods

### Study design

Six PDUs in England were open at the time of study (Goldsmith *et al.*, 2021), four of which had been in operation for more than 2 years allowing sufficient (post-PDU) data for time series analysis. Changes in general and psychiatric hospital activity following the introduction of PDUs in three of these sites (reliable healthcare utilisation data could not be sourced from the other PDU sites) were assessed via a retrospective, secular trend analysis of routinely collected healthcare data using an ITS design. ITS are robust quasi-experimental designs that are increasingly being used to evaluate the impact of changes to healthcare or organisational interventions implemented in healthcare settings where randomisation of the intervention is impractical or unethical (Ewusie *et al.*, 2020). The exposure of interest was implementation of the PDU. The 24 months prior to PDU implementation were considered unexposed, while the 24 months following PDU implementation were exposed. PDU sites were examined individually in the first instance, and then in a pooled analysis to ascertain whether the introduction of PDUs has any overall impact given heterogeneity in service configuration across sites. The methodology of the ITS study has previously been described in detail (Goldsmith *et al.*, 2020).

#### Setting and data collection

Service use data were directly sourced from English psychiatric and general hospitals of PDU sites in two cities and one rural area, according to the timing of the relevant PDU implementation (Urban1: November 2014–November 2018, Urban2: November 2012–November 2016 and Rural: January 2016–December 2019). PDUs were located within psychiatric hospitals that served populations ranging from approximately 750,000 people (Rural) to over a million (Urban1; see Table 1). The EDs were based within general hospitals linked to the psychiatric hospitals, and each supported smaller catchment populations (Urban1: 342,530–364,395, Rural: 414,047–409,228 and Urban2: 392,485–429,986) as, typically, more than one general hospital is located within the catchment area of a psychiatric hospital. Psychiatric hospital data centred on patterns of activity in acute adult (psychiatric) inpatient wards while general hospital data focussed on patterns related to psychiatric presentations in ED. Psychiatric presentations in ED were defined as adult (≥18 years) attendances to a hospital ED where the presenting complaint reflected a mental or behavioural health issue and/or the primary diagnostic code was consistent with a diagnosis of either one or more mental and behavioural disorders (F01–F79 of the International Classification of Diseases, 11th edition) or self-harm (X60–X84) (World Health Organization, 2011). These data were extracted from ‘Presenting Complaint’/‘Reason for Visit’ and ‘Diagnosis’/‘ED Coding’ entries in general hospital ED databases (using the following terms to initially search: %MENTAL%, ‘PSYC&’, ‘SUIC&’, ‘SELF&’ and ‘OVERDOSE’).

**Table 1.** Characteristics of participating psychiatric decision units (PDUs) and service users in first 2 years of operation. Values are frequency (percentage) unless otherwise stated

	Urban1	Rural	Urban2
Time period	November 2014–November 2016	January 2017–December 2019	November 2016–November 2018
Site catchment population	972,372−1010,512	733,663−741,803	848,512−868,334
Structural characteristics			
Capacity	8	6	5
Maximum length of stay	72 (target 24)	24	48
Staff-to-patient ratio	1:4	1:2	1:1
Referral route	CRHT, ED, place of safety team, street triage	AMHPs, CRHT, ED, street triage	CRHT, ED, street triage
Attendances (service users)	2506 (1864)	1793 (1255)	1429 (1006)
Female gender	1227 (49.0)	966 (53.9)	752 (53.2)
Age (mean, SD)	36.1 (12.8)	36.3 (13.6)	37.8 (13.4)
18−24 years	580 (23.1)	428 (23.9)	295 (20.9)
25−64 years	1869 (74.6)	1326 (74.0)	1087 (76.9)
65+ years	57 (2.3)	39 (2.1)	31 (2.2)
Length of stay			
Median hours (IQR)	–	23.0 (10.3,37.7)	37.0 (21.0,48.0)
Discharge to psychiatric hospital	506 (20.2)	237 (13.2)	457 (32.0)

*Notes*: To avoid duplicate admissions for the same event, recorded PDU attendances separated by <12 hours were considered as a single episode (where subsequent visit was <12 hours after discharge only the first episode of the two was considered). Two service users from Urban1 identified as non-binary. Gender and age data for Urban2 were not available for 15 and 16 PDU service users, respectively (percentages are calculated from available data). Where discharge destination was recorded as ‘unknown’, then it was assumed that the individual was not discharged to an acute psychiatric inpatient ward. AMHPs = approved mental health professionals’ teams; CRHT = crisis resolution and (intensive) home treatment team; ED = Liaison Psychiatry/Mental Health Liaison Team in the emergency department; IQR = interquartile range.

Primary outcome measures were the number of voluntary acute adult psychiatric inpatient admissions and number of mental health-related ED attendances. Secondary outcome measures were total (acute adult psychiatric) inpatient admission frequency, proportion of inpatient admissions that were compulsory and length of inpatient stay (both of which may increase if PDU implementation is associated with decreased rate of short-stay voluntary admissions), acute adult ward bed occupancy, frequency of ED-referred psychiatric liaison episodes, length of mental health-related ED stay (LoS), proportion of mental health-related ED stays that breach 4-hour recommended maximum wait times and proportion of mental health-related ED attendances with arrival by ambulance or police. Inpatient admissions were classified as voluntary or compulsory according to the legal status at admission. PDU data (e.g., number of visits and length of visit) pertaining to the first 2 years of operation for each site were also collected. Additionally, strategic managers in each site were spoken to with a view to identify other changes to the crisis-care pathway (e.g., introduction or withdrawal of relevant services).

### Ethical review

The study was registered with and received governance approval from R&D departments of participating NHS Trusts. Approval for the project was granted from the East Midlands Leicester South Research Ethics Committee (19/EM/0226).

#### Statistical analyses

PDU service use parameters were descriptively summarised. Outcome data were collated as time series over a (maximum) 48-month period for each site, aggregated to a single observation at weekly or monthly units. Segmented generalised linear model (GLM) regression analyses (with log or identity link) were employed to evaluate whether there was a change in healthcare utilisation outcomes following PDU implementation (Wagner *et al.*, 2002). This method allowed the calculation of three regression coefficients (with 95% confidence intervals [CIs]) to quantify the impact of service-level change: underlying trend prior to PDU implementation, level change immediately following PDU implementation and slope change from pre-to-post-PDU implementation. Estimates of PDU implementation effect on (inpatient) admissions and mental health-related ED attendances were made by calculating percentage change (immediate and weekly/monthly) from regression coefficients, and where significant step and/or slope changes were observed, by using the pre-PDU model to estimate hypothetical weekly rates of admissions/attendances in the 24 months post-PDU period if no intervention had occurred and calculating the overall (percentage) difference from observed rates (Wagner *et al.*, 2002). To estimate overall effects for primary outcomes, parameter estimates of PDU effect were pooled across sites in a meta-analytical model using an inverse variance approach (Gebski *et al.*, 2012).

In all segmented GLM models, robust (sandwich) variance estimators were applied to account for possible multiple admissions per patient and autoregressive lagged variables fitted as required. Since mental healthcare service utilisation is known to follow a seasonal pattern (Hamilton *et al.*, 2015), Fourier terms based on (trigonometric) sine and cosine functions with a period of 1 year were also included (Lopez-Bernal *et al.*, 2016). In ITS analysis of each site, count data were adjusted for the size of the catchment population supported by the psychiatric hospital (inpatient admissions) or general hospital (mental health-related ED attendances). Additional ITS analyses were conducted for counts of inpatient admissions, mental health-related ED attendances and psychiatric liaison episodes considering only those people who, in the preceding 24 months, had been discharged from psychiatric inpatient services, attended the ED (for any reason) and been referred to psychiatric liaison services, respectively. Secondary analyses of primary outcome measures in ITS were also performed to account for the impact of any other service reconfigurations relevant to outcome measures by introducing a second break-point in ITS models, subject to reconfigurations being sufficiently distant in time from PDU implementation to distinguish any impact.

A *p*-value of less than 0.05 was considered statistically significant in all analyses. Analyses were administered using Stata (StataCorp., Texas, Version 16.1) and SPSS (IBM, Version 26). Further details of data extraction and ITS methods are provided in the Appendix.

## Results

### Structural characteristics and patterns of PDU activity

Urban1, which has the largest capacity and several referral routes, had the highest number of PDU visits (1253.0/year or 125.4/100,000 adult population) while Urban2 had the lowest (714.5/year or 82.8/100,000 adult population). Rural had 896.5 visits annually although the rate per population was comparable with Urban1 (121.5/100,000 adult population). Staff-to-patient ratio was highest in Urban2, which after 1 year of operation included provision of therapeutic input and/or psychosocial help, and lowest in Urban1 which focussed more exclusively on assessment and treatment plan development (Trethewey *et al.*, 2019). Most attendees in each site were aged 25–64 years with an even gender split or small female majority. Length of stay on PDUs was, on average, longer in Urban2 than Rural. Precise LoS was not available for Urban1, although 43.4% of attendees were discharged on the same day as admission and 39.4% on the next day, indicative of the shortest length of stay. The proportion of attendees discharged to psychiatric inpatient wards ranged from 13.2% in Rural to just under a third in Urban2.

### ITS: primary outcomes

In general, voluntary inpatient admissions were considerably greater in Urban1 (848.3/year or 85.8/100,000 adult population) and Urban2 (794.3/year or 92.6/100,000 adult population) than Rural (383.8/year or 52.4/100,000 adult population) with notable proportions of individuals with previous (in last 24 months) inpatient admissions (39.3%, 36.8% and 44.3%, respectively). Voluntary admissions made up about half of all admissions in Urban1 (47.9%) and Urban2 (50.3%) but over 60% of admissions in Rural (61.5%). Similarly, mental health-related attendances in ED were more frequent in Urban1 (2242/year or 592.7/100,000 adult population) and Urban2 (1980/year or 480.3/100,000 adult population) than Rural (1620/year or 393.8/100,000 adult population) with high rates of attendances by service users who had previously attended in the last 24 months (55.1%, 61.1% and 34.4%, respectively).

**Figure 1.**
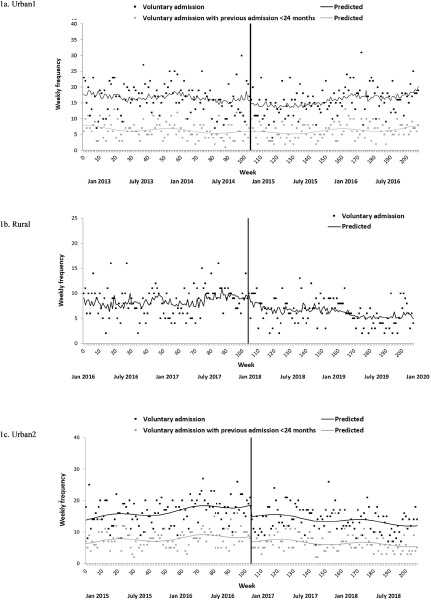
(a, b, c) Impact of PDU implementation on weekly number of voluntary psychiatric inpatient admissions in participating sites. Notes: The black vertical line represents implementation of the PDU. Autoregressive terms were included in voluntary admission models for Urban1 (third-order) and Rural (second-order). Voluntary acute adult psychiatric inpatient admissions in Rural for service users with a previous admission in the last 24 months is considered in monthly aggregated units (Figure A.7).

ITS analyses of weekly aggregated data indicated a significant decrease in the number of voluntary admissions following PDU implementation for Urban1 (−16.2%, CI = −28.4%, −1.8%; Fig. 1a) and Urban2 (−19.6%, CI = −30.0%, −7.5%; Fig. 1c) sites (Table 2). There were significant (long-term) changes in pre-to-post-PDU trend for each site: in the 2-year post-PDU period, the number of voluntary admissions decreased by 0.45%/week in Rural (CI = −0.78%, −0.12%; Fig. 1b) and almost half a percent/week in Urban2 (−0.49%, CI = −0.73%, −0.25%; Fig. 1c) relative to the 2 years prior to PDU implementation. Conversely, they increased by a third of a percent/week in Urban1 (0.33%, CI = 0.07%, 0.59%; Fig. 1a). Comparing the total number of voluntary admissions in the 24 months post-PDU period for each site with the predicted number of admissions without the influence of the PDU indicated that the introduction of the PDU was associated with a 35.3% decrease in voluntary admissions in Rural (*n* = −357), a 37.9% decrease in Urban2 (*n* = −878) and a negligible increase (0.001%) in Urban1 (*n* = +1). Overall, there was a robust pooled level decrease of 16.6% (CI = −23.9%, −8.5%) in voluntary admission frequency across the three participating sites but a non-significant decrease in trend from pre-to-post-PDU (by 0.20%/week, CI = −0.74%, 0.34%; Table 2). A similar pattern of results was observed in analyses considering only admissions by service users with a recent previous admission (Table A.1).

**Table 2.** Changes in level and trend of weekly voluntary inpatient psychiatric admissions and mental health-related ED attendances for participating psychiatric hospitals with meta-analysis

	Initial trend (pre-PDU)	Step change (following PDU)	Trend change (following PDU)
	*B* (95% CI) Weekly change	*B* (95% CI) Step change	*B* (95% CI) Weekly change
Voluntary inpatient admission			
Urban1	−0.001 (−0.003, 0.001)	**−0.176 (−0.334,** −0.018)*	0.003 (0.001, 0.006)*
	−0.07% (−0.26%, 0.13%)	**−16.16% (−28.41%, −1.80%)**	**0.33% (0.07%, 0.59%)**
Rural	0.001 (−0.001, 0.003)	−0.113 (−0.313, 0.086)	−0.004 (−0.008, −0.001)**
	0.09% (−0.13%, 0.31%)	−10.72% (−26.85%, 8.97%)	**−0.45% (−0.78%, −0.12%)**
Urban2	0.003 (0.001, 0.004)**	−0.218 (−0.357, −0.078)**	−0.005 (−0.007, −0.003)***
	**0.27% (0.11%, 0.44%)**	**−19.56% (−30.04%, −7.51%)**	**−0.49% (−0.73%, −0.25%)**
Pooled	0.001(−0.001, 0.003)	−0.181(−0.273, −0.088)***	−0.002 (−0.007, 0.003)
	0.11% (−0.10%, 0.31%)	**−16.56% (−23.94%, −8.45%)**	−0.20% (−0.74%, 0.34%)
Mental health-related ED attendance			
Urban1	0.005 (0.001, 0.008)**	−0.229 (−0.352, −0.105)***	−0.001 (−0.004, 0.002)
	**0.47% (0.14%, 0.79%)**	**−20.43% (−29.70%, −9.95%)**	−0.06% (−0.37%, 0.25%)
Rural	0.0001 (−0.001, 0.001)	0.047 (−0.066, 0.161)	0.003 (0.001, 0.004)**
	0.01% (−0.12%, 0.14%)	4.84% (−6.41%, 17.43%)	**0.26% (0.09%, 0.44%)**
Urban2	−0.001 (−0.002, 0.0005)	0.087 (−0.010, 0.184)	−0.0001 (−0.002, 0.001)
	−0.06% (−0.17, 0.05)	9.12% (−0.96%, 20.22%)	−0.01% (−0.16%, 0.13%)
Pooled	0.0001 (−0.001, 0.003)	−0.029 (−0.214, 0.157)	0.001 (−0.001, 0.003)
	0.08% (−0.12%, 0.28%)	−2.81% (−19.29%, 17.04%)	0.08% (−0.13%, 0.28%)

*Notes:* Time period under study and catchment area population (minimum–maximum during this period) in each site for psychiatric hospital and participating general hospital were as follows: Urban1, November 2012–November 2016, psychiatric hospital 972,372–1010,512, general hospital 342,530–364,395; Rural, January 2016–December 2019, psychiatric hospital 723,227–741,803, general hospital 414,047–409,228 and Urban2, November 2014–November 2018, psychiatric hospital 848,512–868,334, general hospital 392,485–429,986. Urban1 ED attendance data were not available in the first 12 months of the time series.

Asterisks indicate significant changes (emboldened);

* *p* < 0.05, **p < 0.01, ***p < 0.001.

**Figure 2.**
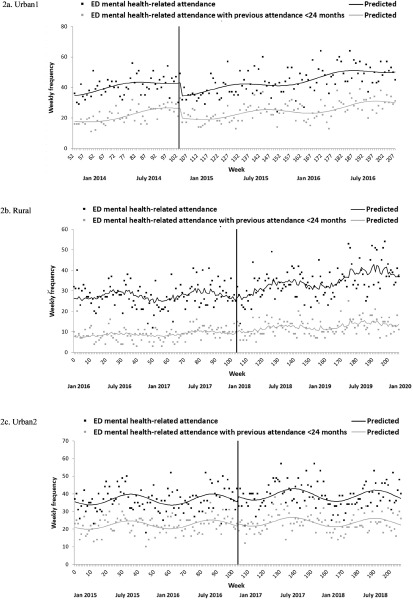
(a, b, c) Impact of PDU implementation on weekly number of mental health-related ED attendances in participating general hospital sites. Notes: The black vertical line represents implementation of the PDU. Autoregressive terms (first-order) were included in ED attendance and ED attendance with previous attendance <24 months models for Rural. Urban1 ED attendance data were not available in the first 12 months of the time series.

The weekly frequency of mental health-related ED attendances in participating general hospitals over the study period is shown graphically in Fig. 2a–c. The number of mental health-related ED attendances decreased significantly following PDU implementation in Urban1 (−20.4%, CI = −29.7%, −10.0%; Table 2), but there was little impact on long-term trend. The pattern was similar when considering only those attendances by service users with a (recent) previous ED attendance (Table A.1), although the initial period was characterised by a more marked weekly increase in frequency (0.79%/week, CI = 0.44%, 1.14%) and there was a significant decrease in trend from pre-to-post-PDU (−0.37%/week, CI = −0.70%, −0.04%). In contrast, while there was no indication of an immediate effect of PDU implementation on mental health-related ED attendances in Rural, there was a significant pre-to-post-PDU trend increase (0.26%/week, CI = 0.09%, 0.44%). Overall, in the 24 months post-PDU period, the introduction of the PDU was associated with a 23.1% decrease in mental health-related ED attendances in Urban1 (*n* = −1374) in contrast to a 24.8% increase in Rural (*n* = +713). Finally, there were no immediate or long-term effects of PDU implementation on mental health-related ED attendances in Urban2. Pooled data analysis suggested that, after adjustment for heterogeneity across sites, implementation of PDUs had no overall effect on level or trend in mental health-related attendances at ED.

### ITS: secondary outcomes

Parameter estimates for secondary outcome measures from ITS analyses of psychiatric and general hospital activity are shown in Table 3 and Table A.2 (corresponding patterns of activity for measures are presented graphically in the Appendix [Figures A.1–A.29]). Both Rural and Urban2 observed highly significant decreases in pre-to-post-PDU trend for all (compulsory and voluntary) inpatient admissions (of 0.5%/week and 0.3%/week, respectively), arresting a prior trend of increasing admission frequency. Comparing admission numbers in the 24 months post-PDU period to that predicted if no intervention had occurred suggested PDU implementation was associated with a 32.1% decrease in inpatient admissions in Rural (*n* = −613) and a 27.0% decrease in Urban2 (*n* = −848). Following PDU implementation, there was an increase in the proportion of compulsory admissions for each site (by 6.3–10.3% depending on site), with a small but reliable weekly increase in Urban2 thereafter. In contrast, the weekly proportion of compulsory admissions in the 2 years post-PDU implementation steadily decreased in Urban1, reflecting a highly significant change in rate from pre-to-post-PDU. The PDU had little impact on length of inpatient stay in any site. Prior to PDU implementation, daily bed occupancy was increasing in Urban1 and Urban2. However, this trend reversed in Urban1 after the implementation of the PDU, reflecting a decrease from pre-to-post-PDU of 0.17 beds/week (Table A.2). In each site, psychiatric liaison episode frequency in the pre-PDU period was significantly increasing (by 0.18–0.24%/week), but only Urban1 observed a significant decrease immediately following PDU implementation (by 10%), and no site evidenced a long-term change (Table A.2).

**Table 3.** Changes in level and trend of (weekly) psychiatric inpatient admissions and mental health-related ED attendance length of stay in participating hospital sites post-PDU implementation

	Initial trend (pre-PDU)	Step change (following PDU)	Trend change (following PDU)
**Urban1**	*B* (95% CI) Weekly change	*B* (95% CI) Step change	*B* (95% CI) Weekly change
Psychiatric inpatient admission			
All inpatient admissions	−0.0001 (−0.001, 0.001)	−0.047 (−0.150, 0.056)	0.001 (−0.001, 0.002)
	−0.01% (−0.13%, 0.10%)	−4.56% (−13.92%, 5.81%)	0.07% (−0.09%, 0.22%)
Compulsory inpatient admission (%)	0.039 (−0.030, 0.108)	6.308 (0.813, 11.803)*	−0.156 (−0.246, −0.067)***
	0.04% points (−0.03%, 0.11%)	**6.31% points (0.81%, 11.80%)**	**−0.16% points (−0.25%. −0.07%)**
Mental health-related ED attendance			
Length of stay: 4-hour breach (%)	0.061 (−0.112, 0.235)	−6.00 (−12.25, 0.247)	−0.014 (−0.187, 0.158)
	0.06% points (0.11%, 0.24%)	−6.00% points (−12.25%, 0.25%)	−0.01% points (−0.19%, 0.16%)
Length of stay: (log) mean minutes	0.0005 (−0.001, 0.002)	−0.121 (−0.202, −0.040)**	0.0001 (−0.002, 0.002)
	0.05% (−0.15%, 0.25%)	**−11.43% (−18.33%, −3.94%)**	0.01% (−0.19%, 0.21%)
**Rural**			
Psychiatric inpatient admission			
All inpatient admissions	0.003 (0.001, 0.005)**	−0.031 (−0.180, 0.119)	−0.004 (−0.007, −0.002)***
	**0.29% (0.10%, 0.49%)**	−3.03% (−16.48%, 12.58%)	**−0.45% (−0.69%, −0.20%)**
Compulsory inpatient admission (%)	0.143 (0.049, 0.236)**	10.275 (2.425, 18.126)*	−0.025 (−0.152, 0.103)
	**0.14% points (0.05%, 0.24%)**	**10.28% points (2.42%, 18.13%)**	−0.03% points (−0.15%, 0.10%)
Mental health-related ED attendance			
Length of stay: 4-hour breach (%)	0.005 (−0.052, 0.063)	1.38 (−3.87, 6.63)	0.032 (−0.045, 0.110)
	0.01% points (−0.05%, 0.06%)	1.38% points (−3.87%, 6.63%)	0.03% points (−0.04%, 0.11%)
Length of stay: (log) mean minutes	−0.0001 (−0.001, 0.001)	0.029 (−0.043, 0.100)	0.001 (0.0002, 0.002)*
	−0.01% (−0.07%, 0.06%)	2.92% (−4.18, 10.56)	**0.12% (0.02%, 0.22%)**
**Urban2**			
Psychiatric inpatient admission			
All inpatient admissions	0.002 (0.001, 0.003)***	−0.089 (−0.184, 0.007)	−0.003 (−0.004, −0.001)***
	**0.22% (0.12%, 0.32%)**	−8.50% (−16.83%, 0.66%)	**−0.28% (−0.43%, −0.13%)**
Compulsory inpatient admission (%)	−0.036 (−0.098, 0.026)	6.85 (1.685, 12.017)**	0.109 (0.021, 0.197)*
	−0.04% points (−0.10, 0.03%)	**6.85% points (1.69%, 12.02%)**	**0.11% points (0.02%, 0.20%)**
Mental health-related ED attendance			
Length of stay: 4-hour breach (%)	−0.035 (−0.090, 0.020)	2.576 (−2.116, 7.268)	0.075 (−0.003, 0.153)
	−0.04% points (−0.09%, 0.02%)	2.58% points (−2.12%, 7.27%)	0.08% points (−0.003%, 0.16%)
Length of stay: (log) mean minutes	−0.001 (−0.002, −0.0002)*	0.006 (−0.066, 0.078)	0.003 (0.001, 0.004)***
	**−0.11% (−0.20%, −0.02%)**	0.59% (−6.36%, 8.06%)	**0.27% (0.14%, 0.39%)**

*Notes*: Psychiatric liaison episode data were not available in the first 6 months of the time series in Rural.

Asterisks indicate significant changes (emboldened);

* *p* < 0.05, **p < 0.01, ***p < 0.001.

There was no significant impact of PDU implementation on the proportion of mental health-related ED attendances via ambulance/police (Table A.2) or rates of 4-hour breaches in any site (Table 3). There was, however, a significant drop (>10%) in LoS immediately following PDU implementation in Urban1. Conversely, weekly average ED LoS increased post-PDU (relative to pre-PDU) in both Rural and Urban2.

ITS analyses that (separately) included each major service initiative in conjunction with PDU implementation tended to reaffirm the significant decrease in informal inpatient admissions following PDU implementation (Tables A.3–A.5, Appendix Results). Other initiatives significantly affected short-term admission rates also, however, such as the (pre-PDU) introduction of street triage services in Urban1 (decrease of 20.5%) and the opening of a psychiatric intensive care unit (PICU) in Rural (increase of 34.7%). Long-term weekly admission rates in Urban2 also reduced following the introduction of crisis café (20 weeks post-PDU) and street triage services (30 weeks post-PDU). Implementation of other service-level initiatives had less impact on ED mental health-related attendances.

## Discussion

This is the first quasi-experimental analysis of PDU impact on the crisis-care pathway in England. The findings suggest that PDUs reduced overall levels of voluntary acute psychiatric admissions after their implementation (by about 17%). This is consistent with previous research examining short-stay mental health crisis units, which found proportions of ED patients who experienced a psychiatric admission after opening of short-stay mental health crisis units reduced by 7–17% across studies (Anderson *et al.*, 2022; Lester *et al.*, 2018; Parwani *et al.*, 2018; Stamy *et al.*, 2021). Two sites under study also observed reliable long-term decreases in the weekly rate of voluntary psychiatric admissions accounting for pre-exposure and seasonal trends, suggesting PDU implementation was associated with a 35–40% reduction in total voluntary admissions in each site over the post-PDU period (based on comparisons with estimates that followed pre-PDU admission patterns). In both cases, reductions were also observed considering all (voluntary and compulsory) psychiatric admissions, arresting the increasing weekly trend prior to PDU implementation. This suggests a net change (estimated decrease of 27–33%) in the pattern of overall inpatient service use over an extended period, and more specifically, that decreasing rates of voluntary admissions in these sites is unlikely to reflect a broader (20-year) trend of declining admission rates in England (Degli Esposti *et al.*, 2022) or an inevitable consequence of increasing numbers of formal detentions in psychiatric hospitals observed across England over recent years (Rains *et al.*, 2019). Conversely, the other (urban) site observed a significant increase in weekly voluntary admission rate in the 24-month period post-PDU implementation (which cancelled out a reliable short-term reduction), although there was no discernible change in rate of all psychiatric admissions.

The PDUs associated with decreased (voluntary) psychiatric admissions over the long-term (Rural, Urban2) had less throughput, longer lengths of stay (up to 40 hours on average) and higher staff-to-patient ratios than the PDU that did not (Urban1), and enabled some therapeutic input (e.g., delivery of psychosocial interventions). As such, these units are well placed to improve service user experience of crisis care, particularly for individuals with a high level of crisis need who do not necessarily benefit from extensive inpatient care (e.g., people with diagnoses of personality disorders or complex emotional needs and/or people who have self-harmed) (Stulz *et al.*, 2015), consistent with findings from comparable PES units internationally (Lester *et al.*, 2018; Parwani *et al.*, 2018). Significant long-term decreases in voluntary admission frequency were also observed in these two sites (Rural, Urban2) when considering only those service users with a recent admission, suggesting that people at high risk of psychiatric admission can also avoid admission via PDU attendance.

The lack of (overall) impact on parameters linked to ED mental health-related attendances was surprising, especially considering the success of short-stay mental health crisis units or PES outside the UK (with broadly comparable configurations) in decreasing both the frequency of ED presentations and the average length of ED stay (Anderson *et al.*, 2022; Braitberg *et al.*, 2018; Lester *et al.*, 2018; Parwani *et al.*, 2018; Stamy *et al.*, 2021). Only one of the three PDUs examined successfully reduced mental health-related attendances at ED; PDU implementation at Urban1 induced a short-term reduction in attendances by 20% and a 10% decrease in the number of psychiatric liaison episodes, effects that were heightened in frequent users of each (23% and 15% decreases, respectively). There was also an 11% drop in average length of ED stay after PDU implementation in this site. This PDU had the highest throughput, shortest average length of stay and lowest staff-to-patient ratio. Although stays at this PDU are relatively short (median <9 hours (Trethewey *et al.*, 2019)), the PDU appeared to be successful in diverting service users away from the suboptimal assessment environment of the ED; many of these service users had diagnoses of personality or mood disorders, both of which are linked to particularly negative experiences of ED by affected individuals (Digel Vandyk *et al.*, 2018) and for whom ED staff find appropriate assessment and management challenging (Dombagolla *et al.*, 2019).

Overall, when examined using quasi-experimental methods, the benefits of PDUs on either voluntary psychiatric admissions or mental health-related attendances at ED appear to be closely linked to unit configuration and site priorities (based on pre-PDU trends). This is consistent with findings from a recent systematic review on comparable units internationally, which suggested that units with a clear purpose and configuration to impact ED wait times are effective in doing so, and units that primarily aim and are configured to reduce psychiatric admissions are effective in doing so (Anderson *et al.*, 2022). Whether the advantages of the different PDU models yield cost-benefits to participating sites is yet to be established. Potential savings are, in part, dependent on shorter length of psychiatric inpatient stays (Mason *et al.*, 2011), and there were no (short- or long-term) effects of observed on inpatient LoS or bed occupancy in either of the PDU sites with long-term reductions in voluntary admission rates (Rural, Urban2). This is perhaps unsurprising given the increased proportion of compulsory admissions post-PDU at these sites, which are associated with longer inpatient stays (Jacobs *et al.*, 2015). Further, in the short term, use of PDUs leads to increased service use (and increased associated costs) via signposting to community resources, at least for first-time PDU visitors, although encouragingly this does not reflect an increase in ED attendances (Goldsmith *et al.*, 2022). Also, there was little evidence to suggest a long-term change to the backdrop of (week-to-week) increasing frequency of ED presentations and/or psychiatric liaison episodes observed across sites (and seen across hospitals in England (Baracaia *et al.*, 2020; NHS England, 2021)). EDs in England have experienced continued increase in demand (NHS England, 2021) and as such the PDUs in our study might be functioning to mitigate that additional demand, rather than decreasing use of ED as seen in the international literature.

There are some limitations to this ITS study. First, although data were extracted directly from participating NHS Trusts, enabling measurement of many parameters, we nevertheless relied on routinely collected data. Mental health diagnostic coding can be particularly problematic in datasets derived from general hospital data (Davis *et al.*, 2016), and any changes in coding practice over the study period complicate interpretation of trends. In two (urban) sites, data concerning ED-based activity were only available for one of the two general hospitals linked to the PDUs, and for a small number of variables datasets did not cover the entire 4-year period of interest; this decreased the power of corresponding ITS analyses. Data pertaining to out-of-area (inpatient) NHS placements (which are commonly attributed to high levels of mental health bed occupancy and incur considerable expense (Wyatt *et al.*, 2019)) and use of private hospitals were not available, limiting the scope of investigation. Additionally, there were a number of secondary outcome measures giving rise to several ITS analyses, each administered without correction for multiple testing.

Finally, while pooled ITS analyses investigating whether there was a significant effect overall (across sites) addresses an important question at a (NHS) policy level, that is, does the introduction of PDUs have an overall impact given the variation in configuration, interpretation of findings is complicated by the effect of broader (national) conditions that may have differed across sites as the years under study were distinct. Local conditions outside the hospital settings of each site may have also impacted observed trends. For example, reduced availability and/or quality of community mental health services have been linked with higher rates of compulsory psychiatric admissions in England (Rains *et al.*, 2020).

## Conclusion

Internationally, observational studies of short-stay mental health crisis units have reported benefits with respect to ED and psychiatric hospital activity. The present study adopted robust quasi-experimental methods and found that, overall, the impact of the emergent models of PDUs in England on psychiatric inpatient admissions and psychiatric presentations in EDs is modest. However, the PDUs under study were not set up in a uniform manner, and analyses of individual sites indicated benefits of each that were closely linked to unit configuration, suggesting that PDUs can be configured to best address the primary crisis-care need locally, whether that be to reduce either unhelpful or avoidable voluntary psychiatric admissions, or relieving pressure on ED. Implementation of PDUs often occurred as part of or concurrently with reconfiguration or expansion of crisis-care services at participating sites. While the present study demonstrated some success both in isolating PDU-specific effects (decrease in voluntary psychiatric admissions) and identifying complementary effects of other service-led initiatives (such as the opening of PICU and/or crisis cafes/houses), future work exploring how PDUs can optimally interlink with other inputs into the mental health crisis-care pathway is needed, to better elucidate impact on patterns of general and psychiatric hospital activity and inform corresponding policy and commissioning.

## Supporting information

Smith et al. supplementary material 1 Smith et al. supplementary material

Smith et al. supplementary material 2 Smith et al. supplementary material

## Data Availability

The data that support the findings of this study are available from the corresponding author, upon reasonable request, to researchers who provide a methodologically sound proposal. Proposals should be directed to the corresponding author; to gain access, data requestors will need to sign a data access agreement.

## Supplementary material

The supplementary material for this article can be found at https://doi.org/10.1017/S2045796024000209.
