# The effect of psychiatric decision unit services on inpatient admissions and mental health presentations in emergency departments: an interrupted time series analysis from two cities and one rural area in England – CORRIGENDUM

**DOI:** 10.1017/S2045796024000271

**Published:** 2024-04-12

**Authors:** J. G. Smith, K. Anderson, G. Clarke, C. Crowe, L. P. Goldsmith, H. Jarman, S. Johnson, J. Lomani, D. McDaid, A. Park, K. Turner, S. Gillard

DOI: https://doi.org/10.1017/S2045796024000209, Published by Cambridge University Press, 21 March 2024

Author name A. Park was incorrect in the original publication of the article. This has been corrected and republished.

The authors apologize for the error.
