# Supplementary material for: The effect of psychiatric decision unit services on inpatient admissions and mental health presentations in emergency departments: an interrupted time series analysis from two cities and one rural area in England
Source: Epidemiol Psychiatr Sci. 2024 Mar 21;33:e15. doi: 10.1017/S2045796024000209 (PMC11362677; doi:10.1017/S2045796024000209)
Supplement: Smith et al. supplementary material 1 — Smith et al. supplementary material [file S2045796024000209sup001.docx]

**Supplementary appendix**

Supplementary tables and figures

| **Table A.1.** Changes in level and trend of weekly voluntary psychiatric inpatient admissions, mental health-related ED attendances and (ED-based) liaison psychiatry episodes of frequent service users in participating psychiatric and general hospital sites after introduction of a PDU. | | | |
| --- | --- | --- | --- |
|  | Initial trend  (Pre-PDU) | Step change  (Following PDU) | Trend change  (Following PDU) |
| **Urban1** | B (95% CI)  Weekly change | B (95% CI)  Step change | B (95% CI)  Weekly change |
| Voluntary inpatient admission  (with admission previous ≤24 mths) | -.003(-.005,.0001) | -.052(-.286,.182) | **.005(.002,.009)**** |
|  | -0.26% (-0.52%,0.01%) | -5.06% (-24.89%,20.01%) | **0.53% (0.18%,0.88%)** |
| ED mental health attendance  (with attendance previous ≤24 mths) | **.008(.004,.011)***** | **-.268(-.406,-.129)***** | **-.004(-.007,-.0004)*** |
|  | **0.79% (0.45%,1.14%)** | **-23.48% (-33.35%,-12.14%)** | **-0.37% (-0.70%,-0.04%)** |
| Psychiatric liaison episode  (with episode previous ≤24 mths) | **.005(.004,.006)***** | **-.158(-.226,-.090)***** | **-.003(-.004,-.002)***** |
|  | **0.48% (0.39%,0.56%)** | **-14.58% (-20.21%,-8.56%)** | **-0.28% (-0.39%,-0.18%)** |
| **Rural** | Monthly change | Step change | Monthly change |
| Voluntary inpatient admission  (with admission previous ≤24 mths) | .010(-.008,.028) | .036(-.264,.335) | **-.030(-.053,-.007)*** |
|  | 1.00% (-0.83%,2.86%) | 3.64% (-23.17%,39.81%) | **-2.96% (-5.17%,-0.70%)** |
|  | Weekly change | Step change | Weekly change |
| ED mental health attendance  (with attendance previous ≤24 mths) | .002(-.001,.004) | .095(-.084,.274) | .0005(-.003,.004) |
|  | 0.18% (-0.07%,0.43%) | 9.98% (-8.04%,31.54%) | 0.05% (-0.25%,0.35%) |
|  | Monthly change | Step change | Monthly change |
| Psychiatric liaison episode  (with episode previous ≤24 mths) | **.039(.027,.051)***** | -.002(-.187,.183) | **-.028(-.043,-.014)***** |
|  | **3.97% (2.70%,5.26%)** | -0.22% (-17.07%,20.06%) | **-2.79% (-4.21%,-1.35%)** |
| **Urban2** | Weekly change | Step change | Weekly change |
| Voluntary inpatient admission  (with admission previous ≤24 mths) | **.003(.001,.005)*** | **-.219(-.402,-.037)*** | **-.005(-.008,-.002)***** |
|  | **0.28% (0.06%,0.50%)** | **-19.70% (-33.11%,-3.60%)** | **-0.53% (-0.84%,-0.23%)** |
| ED mental health attendance  (with attendance previous ≤24 mths) | -.0003(-.002,.001) | .062(-.051,.175) | -.0004(-.002,.001) |
|  | -0.02% (-0.15%,0.10%) | 6.43% (-4.94%,19.16%) | -0.04% (-0.23%,0.14%) |
| Psychiatric liaison episode  (with episode previous ≤24 mths) | **.003(.002,.004)***** | .075(-.034,.184) | -.001(-.003,.0003) |
|  | **0.30% (0.18%,0.43%)** | 7.79% (-3.30%,20.15%) | -0.15% (-0.32%,0.03%) |
| Notes: mths = months Asterisks indicate significant changes (emboldened); **p*<0.05,***p*<0.01,****p*<0.001. Comparing the number of voluntary admissions (for those with previous admission) in the 24-month post-PDU period with the predicted number of admissions without the influence of the PDU indicated that the introduction of the PDU was associated with a 28.8% decrease in voluntary admissions in Rural (n=-107), a 39.3% decrease in Urban2 (n=-441), and a 25.2% increase in Urban 1 (n=+129). Comparing the number of mental health-related ED attendances (for those with previous attendance) in the 24-month post-PDU period with the predicted number of attendances without the influence of the PDU suggested that PDU implementation was associated with a 38.4% decrease in attendances in Urban1 (n=-1592). Comparing the number of ED-referred psychiatric liaison episodes (for those with previous episode) in the 24-month post-PDU period with the predicted number of episodes without the influence of the PDU indicated that the introduction of the PDU was associated with a 27.2% decrease in psychiatric liaison episodes in Urban1 (n=-2924) and a 31.9% decrease in Rural (n=-670). | | | |

| **Table A2.** Changes in level and trend of (weekly) psychiatric inpatient admissions, acute adult psychiatric bed occupancy, liaison psychiatry activity and arrival patterns for mental health-related ED attendances in participating hospital sites post-PDU implementation. | | | |
| --- | --- | --- | --- |
|  | Initial trend  (Pre-PDU) | Step change  (Following PDU) | Trend change  (Following PDU) |
| **Urban1** | B (95% CI)  Weekly change | B (95% CI)  Step change | B (95% CI)  Weekly change |
| Psychiatric inpatient admission | |  |  |
| Five days or less inpatient admission (%) | .003(-.030,.036) | -.924(-3.729,1.881) | .020(-.028,.069) |
|  | 0.003% points (-0.03%,0.04%) | -0.92% points (-3.73%,1.88%) | 0.02% points (-0.03%,0.07%) |
| Length of stay (log mean days) | .002(-.0003,.004) | -.170(-.369,.029) | -.001(-.004,.002) |
|  | 0.21% (-0.03%,0.45%) | -15.63% (-30.88%,2.96%) | -0.11% (-0.44%,0.21%) |
| Daily bed occupancy (mean) | **.147(.073,.221)***** | -1.248(-4.049,1.554) | **-.171(-.252,-.090)***** |
|  | **0.15 beds (0.07%,0.22%)** | -1.25 beds (-4.05%,1.55%) | **-0.17 beds (-0.25%,-0.09%)** |
| Psychiatric liaison episodes (ED-referred) | **.002(.002,.003)***** | **-.108(-.159,-.056)***** | -.0004(-.001,.0003) |
|  | **0.24% (0.19%,0.30%)** | **-10.22% (-14.71%,-5.48%)** | -0.05% (-0.13%,0.03%) |
| Mental health-related ED attendance | |  |  |
| Arrival by ambulance/police (%) | .010(-.141,.161) | -1.818(-7.169,3.533) | -.005(-.155,.145) |
|  | 0.01% points (-0.14%,0.16%) | -1.82% points (-7.17,3.53%) | -0.01% points (-0.16%,0.15%) |
| **Rural** |  |  |  |
| Psychiatric inpatient admission | |  |  |
| Five days or less inpatient admission (%) | **.085(.016,.154)*** | -2.502(-8.444,3.441) | -.064(-.157,.029) |
|  | **0.09% points (0.02%,0.15%)** | -2.50% points (-8.44%,3.44%) | -0.06% points (-0.16%,0.03%) |
| Length of stay (log mean days) | -.003(-.007,.001) | .083(-.243,.409) | .001(-.004,.006) |
|  | -0.27% (-0.67%,0.14%) | 8.64% (-21.56%,50.46%) | 0.12% (-0.38%,0.62%) |
| Daily bed occupancy (mean) | .006(-.007,.018) | .938(-.252,2.13) | -.014(-.031,.004) |
|  | 0.01 beds (-0.01,0.02) | 0.94 beds (-0.25,2.13) | -0.01 beds (-0.03,0.004%) |
| Psychiatric liaison episodes (ED-referred) | **.002(.0003,.003)*** | .005(-.104,.114) | .001(-.001,.003) |
|  | **0.18% (0.03%,0.34%)** | 0.51% (-9.85%,12.07%) | 0.13% (-0.08%,0.33%) |
| Mental health-related ED attendance | |  |  |
| Arrival by ambulance/police (%) | **-.074(-.132,-.016)*** | 1.702(-3.693,7.097) | .036(-.040,.111) |
|  | **-0.07% points (-0.13%,-0.02%)** | 1.70% points (-3.69%,7.10%) | 0.04% points (-0.04,0.11%) |
| **Urban2** |  |  |  |
| Psychiatric inpatient admission | |  |  |
| Five days or less inpatient admission (%) | -.044(-.093,.005) | 1.110(-3.478,5.697) | -.005(-.076,.065) |
|  | -0.04% points (-0.09%,0.005%) | 1.11% points (-3.48%,5.70%) | -0.01% points (-0.08%,0.07%) |
| Length of stay (log mean days) | -.002(-.005,.0003) | 0.128(-.059,.315) | .002(-.001,.005) |
|  | -0.21% (-0.45%,0.04%) | 13.66% (-5.74%,37.04%) | 0.24% (-0.07%,0.55%) |
| Daily bed occupancy (mean) | **.035(.001,.068)*** | -1.234(-3.492,1.024) | -.029(-.069,.010) |
|  | **0.04 beds (0.001,0.07)** | -1.23 beds (-3.49%,1.02%) | -0.03 beds (-0.07%,0.01%) |
| Psychiatric liaison episodes (ED-referred) | **.002(.001,.003)***** | .018(-.050,.087) | -.001(.002,.0003) |
|  | **0.23% (0.15%,0.32%)** | 1.86% (-4.86%,9.05%) | -0.07% (-0.18%,0.03%) |
| Mental health-related ED attendance | |  |  |
| Arrival by ambulance/police (%) | .014(-.042,.070) | -3.215(-8.000,1.570) | -.067(-.140,.006) |
|  | 0.01% points (--0.04%,0.07%) | -3.22% points (-8.00%,1.57%) | -0.07% points (-0.14%, 0.01%) |
| Notes: Asterisks indicate significant changes (emboldened); **p*<0.05,***p*<0.01,****p*<0.001. Psychiatric liaison episode data was not available in the first 6 months of the time series in Rural. Comparing the number of ED-referred psychiatric liaison episodes (for those with previous episode) in the 24-month post-PDU period with the predicted number of episodes without the influence of the PDU indicated that PDU implementation was associated with a 12.6% decrease in psychiatric liaison episodes in Urban1 (n=-2294). | | | |

| **Table A.3.** Changes in level and trend of primary ITS outcomes in Urban 1 post-PDU implementation considering other changes to the crisis care pathway in the study period. | | | | | |
| --- | --- | --- | --- | --- | --- |
| B (95% CI)  Weekly % change | B (95% CI)  Step % change | B (95% CI)  Weekly % change | B (95% CI)  Weekly % change | B (95% CI)  Step % change | B (95% CI)  Weekly % change |
| **Informal inpatient admissions** | | | | | |
| Initial trend  (Pre-street triage) | Step change  (Post-street triage) | Trend change (Post-street triage) | Step change  (Post-PDU) | Trend change (Post-PDU) | Post-PDU trend |
| .003(-.001,.007) | **-.229(-.449,-.009)*** | -.001(-.010,.008) | -.208(-.453,.037) | .001(-.007,.009) | **.003(.001,.005)***** |
| 0.31% | **-20.50%** | -0.12% | -18.77% | 0.09% | **0.30%** |
| Initial trend  (Pre-PDU) | Step change  (Post-PDU) | Trend change (Post-PDU) | Step change  (Post-new model) | Trend change (Post-new model) | Post-new model trend |
| -.001 (-.003,.001) | **-.234(-.457,-.012)*** | .005(-.001,.012) | -.023(-.230,.184) | -.003(-.010,.003) | **.006(.002,.010)**** |
| -0.06% | **-20.89%** | 0.55% | -2.28% | -0.33 | **0.63** |
| Initial trend  (Pre-PDU) | Step change  (Post-PDU) | Trend change (Post-PDU) | Step change  (Post-PDU expand) | Trend change (Post-PDU expand) | Post-PDU expand trend |
| -.001(-.003,.001) | **-.206(-.397,-.015)*** | **.004(.0001,.009)*** | .065(-.164,.293) | -.005(-.012,.002) | .006(.018,.973) |
| -0.08% | **-18.61%** | **0.44%** | 6.68% | -0.52% | 0.61% |
| **ED mental health-related attendances** | | | | | |
| Initial trend  (Pre-PDU) | Step change  (Post-PDU) | Trend change (Post-PDU) | Step change  (Post-new model) | Trend change (Post-new model) | Post-new model trend |
| **.005(.001,.008)*** | **-.234(-.391,-.076)**** | -.0002(-.005,.004) | .021(-.140,.181) | -.001(-.005,.003) | **.004(.001,.006)**** |
| **0.45%** | **-20.84%** | -0.02% | 2.12% | -0.10% | **0.36%** |
| Initial trend  (Pre-PDU) | Step change  (Post-PDU) | Trend change (Post-PDU) | Step change  (Post-PDU expand) | Trend change (Post-PDU expand) | Post-PDU expand trend |
| **.004 (.001,.008)*** | **-.224(-.362,-.087)**** | -.0002(-.004,.003) | .032(-.124,.189) | -.002(-.006,.003) | .004(-.003,.012) |
| **0.44%** | **-20.09%** | -0.02% | 3.29% | -0.15% | 0.44% |
| ‘New model’ refers to the introduction of a new model of service care intended to re-organise community care is proposed. **p*<0.05, ***p*<0.01, ****p*<0.001. | | | | | |

| **Table A.4.** Changes in level and trend of primary ITS outcomes in Rural post-PDU implementation considering other changes to the crisis care pathway in the study period. | | | | | |
| --- | --- | --- | --- | --- | --- |
| B (95% CI)  Weekly % change | B (95% CI)  Step % change | B (95% CI)  Weekly % change | B (95% CI)  Step % change | B (95% CI)  Weekly % change | B (95% CI)  Weekly % change |
| **Informal inpatient admissions** | | | | | |
| Initial trend  (Pre-PICU open) | Step change  (Post – PICU open) | Trend change (Post – PICU open) | Step change  (Post-PDU) | Trend change (Post-PDU) | Post-PDU trend |
| -.0004(-.004,.003) | **.297(.007,.588)*** | -.011(-.027,.005) | -.035(-.307,.237) | .008(-.008,.024) | **-.005(-.007,-.002)**** |
| -0.04% | **34.65%** | -1.10% | -3.44% | 0.80% | **-0.45%** |
| Initial trend  (Pre-PDU) | Step change  (Post-PDU) | Trend change (Post-PDU) | Step change  (Post-PDU staff change) | Trend change (Post-PDU staff change) | Post-PDU staff change |
| .001(-.001,.003) | -.182(-.479,.115) | -.002(-.017,.013) | .047(-.329,.424) | -.004(-.020,.011) | -.002(-.005,.001) |
| 0.10% | -16.66% | -0.21% | 4.84% | -0.43% | -0.22% |
| Initial trend  (Pre-PDU) | Step change  (Post-PDU) | Trend change (Post-PDU) | Step change  (Post-CVR launch) | Trend change (Post-CVR launch) | Post-CVR launch |
| .001(-.001,.003) | **-.231(-.451,-.010)*** | -.0004(-.005,.004) | **-.513(-.821,-.204)**** | .007(-.005,.020) | -.001(-.024,.021) |
| 0.11% | **-20.60%** | -0.04% | **-40.10%** | 0.75% | -0.13% |
| **ED mental health-related attendances** | | | | | |
| Initial trend  (Pre-PICU open) | Step change  (Post – PICU open) | Trend change (Post – PICU open) | Step change  (Post-PDU) | Trend change (Post-PDU) | Post-PDU trend |
| -.001(-.003,.001) | .007(-.208,.222) | .008(-.003,.001) | -.070(-.224,.085) | -.004(-.015,.007) | **.003(.001,.004)***** |
| -0.11% | 0.70% | 0.78% | -6.73% | -0.38% | **0.27%** |
| Initial trend  (Pre-PDU) | Step change  (Post-PDU) | Trend change (Post-PDU) | Step change  (Post-PDU staff change) | Trend change (Post-PDU staff change) | Post-PDU staff change |
| .0001(-.001,.001) | .033(-.154,.220) | .004(-.003,.010) | -.022(-.159,.115) | -.001(-.008,.006) | **.003(.001,.005)*** |
| 0.01% | 3.33% | 0.35% | -2.20% | -0.08% | **0.27%** |
| Initial trend  (Pre-PDU) | Step change  (Post-PDU) | Trend change (Post-PDU) | Step change  (Post-CVR launch) | Trend change (Post-CVR launch) | Post-CVR launch |
| .0001(-.001,.001) | .057(-.081,.195) | .002(-.001,.005) | -.007(-.164,.150) | .001(-.004,.007) | -.005(-.017,.007) |
| 0.01% | 5.84% | 0.23% | -0.69% | 0.14% | -0.48% |
| PICU = Psychiatric Intensive Care Unit; CVR = Crisis Vehicular Response service. **p*<0.05, ***p*<0.01, ****p*<0.001. | | | | | |

| **Table A.5.** Changes in level and trend of primary ITS outcomes in Urban 2 post-PDU implementation considering other changes to the crisis care pathway in the study period. | | | | | |
| --- | --- | --- | --- | --- | --- |
| B (95% CI)  Weekly % change | B (95% CI)  Step % change | B (95% CI)  Weekly % change | B (95% CI)  Weekly % change | B (95% CI)  Step % change | B (95% CI)  Weekly % change |
| **Informal inpatient admissions** | | | | | |
| Initial trend  (Pre-PDU) | Step change  (Post-PDU) | Trend change (Post-PDU) | Step change  (Post-CC launch) | Trend change (Post-CC launch) | Post-CC launch |
| **.003(.001,.004)***** | **-.589(-.809,-.368)***** | **.020(.001,.039)*** | .010(-.226,.246) | **-.027(-.046,-.008)**** | **-.005(-.007,-.002)***** |
| **0.28%** | **-44.49%** | **2.03%** | 1.03% | **-2.70%** | **-0.50%** |
| Initial trend  (Pre-PDU) | Step change  (Post-PDU) | Trend change (Post-PDU) | Step change  (Post-Street triage launch) | Trend change (Post-Street triage launch) | Post-Street triage launch |
| **.003(.001,.004)***** | **-.552(-.742,-.363)***** | **.014(.005,.023)**** | -.162(-.346,.022) | **-.021(-.030,-.012)***** | **-.004(-.007,-.001)**** |
| **0.29%** | **-42.44%** | **1.43%** | -14.95% | **-2.11%** | **-0.42%** |
| Initial trend  (Pre-PDU) | Step change  (Post-PDU) | Trend change (Post-PDU) | Step change  (Post-PDU expands) | Trend change (Post-PDU expands) | Post-PDU expands |
| .003(.001,.005)*** | **-.386(-.542,-.229)***** | .001(-.003,.005) | **-.357(-.562,.-152)***** | -.002(-.010,.005) | **.012(.001,.023)*** |
| 0.31% | **-31.99%** | 0.10% | **-30.03%** | -0.24% | **0.12%** |
| **ED mental health-related attendances** | | | | | |
| Initial trend  (Pre-PDU) | Step change  (Post-PDU) | Trend change (Post-PDU) | Step change  (Post-CC launch) | Trend change (Post-CC launch) | Post-CC launch |
| -.001(-.002,.001) | .006(-.166,.179) | .002(-.010,.013) | .088(-.033,.210) | -.003(-.014,.009) | **-.002(-.003,-.0001)*** |
| -0.05% | 0.65% | 0.18% | 9.25% | -0.29% | **-0.15%** |
| Initial trend  (Pre-PDU) | Step change  (Post-PDU) | Trend change (Post-PDU) | Step change  (Post-Street triage launch) | Trend change (Post-Street triage launch) | Post-Street triage launch |
| -.001(-.002,.0005) | -.050(-.203,.104) | **.010(.003,.017)**** | **-.182(-.312,-.052)**** | **-.010(-.017,-.002)*** | -.0004(-.002,.001) |
| -0.06% | -4.85% | **0.99%** | **-16.65%** | **-0.96%** | -0.04% |
| Initial trend  (Pre-PDU) | Step change  (Post-PDU) | Trend change (Post-PDU) | Step change  (Post-PDU expands) | Trend change (Post-PDU expands) | Post-PDU expands |
| -.0003(-.001,.001) | .002(-.111,.116) | .003(-.0002,.005) | **-.309(-.444,-.175)***** | **.004(.0001,.008)*** | .004(-.001,.009) |
| -0.03% | 0.23% | 0.25% | **-26.61%** | **0.42%** | 0.41% |
| CC = crisis care café. **p*<0.05, ***p*<0.01, ****p*<0.001. | | | | | |

**
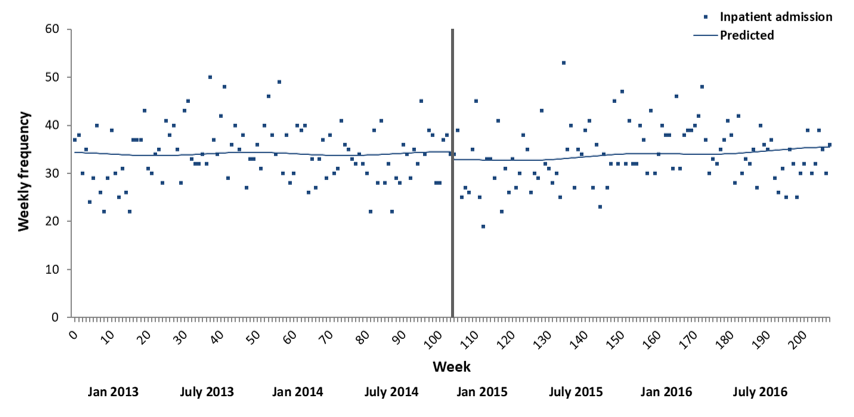
**

**Figure A.1.** The impact of PDU implementation on acute adult psychiatric inpatient admissions in Urban1. Please note: the black vertical line represents the interruption (i.e., implementation of the PDU).

**
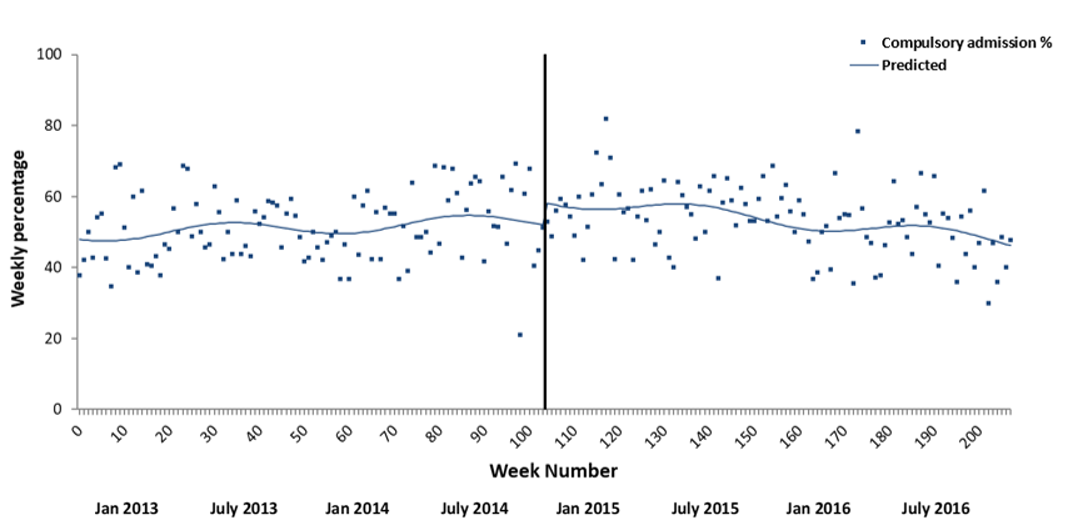
**

**Figure A.2.** The impact of PDU implementation on percentage of compulsory acute adult psychiatric inpatient admissions in Urban1. Please note: the black vertical line represents the interruption (i.e., implementation of the PDU).

**
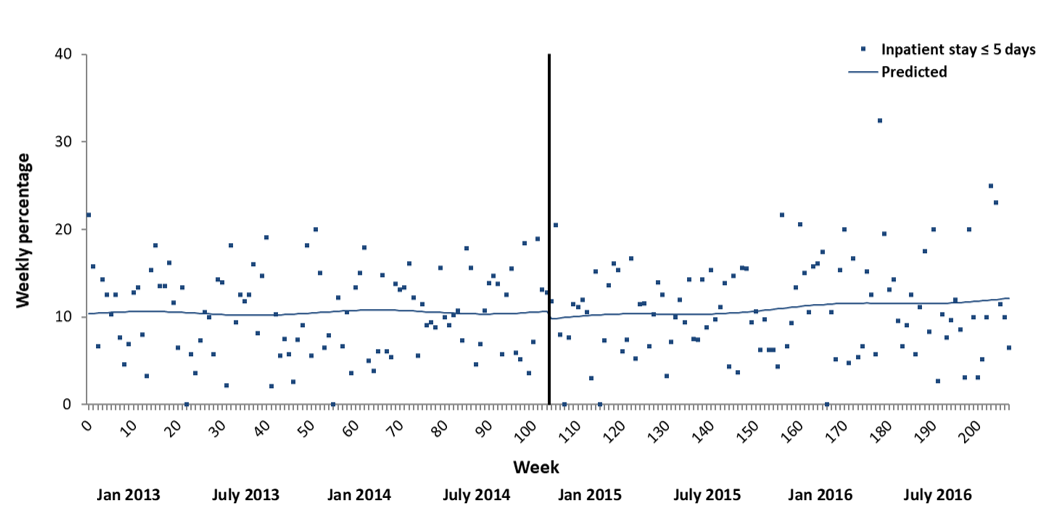
**

**Figure A.3.** The impact of PDU implementation on percentage of acute adult inpatient stays of 0-5 days in Urban1. Please note: the black vertical line represents the interruption (i.e., implementation of the PDU).

**
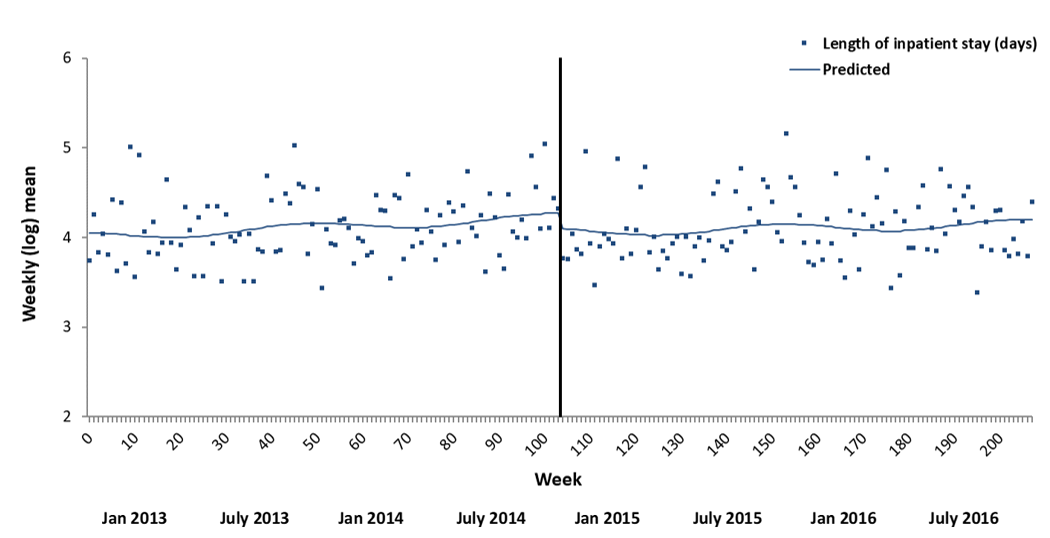
**

**Figure A.4.** The impact of PDU implementation on (log) mean length of acute adult inpatient stays in Urban1. Please note: the black vertical line represents the interruption (i.e., implementation of the PDU).

**
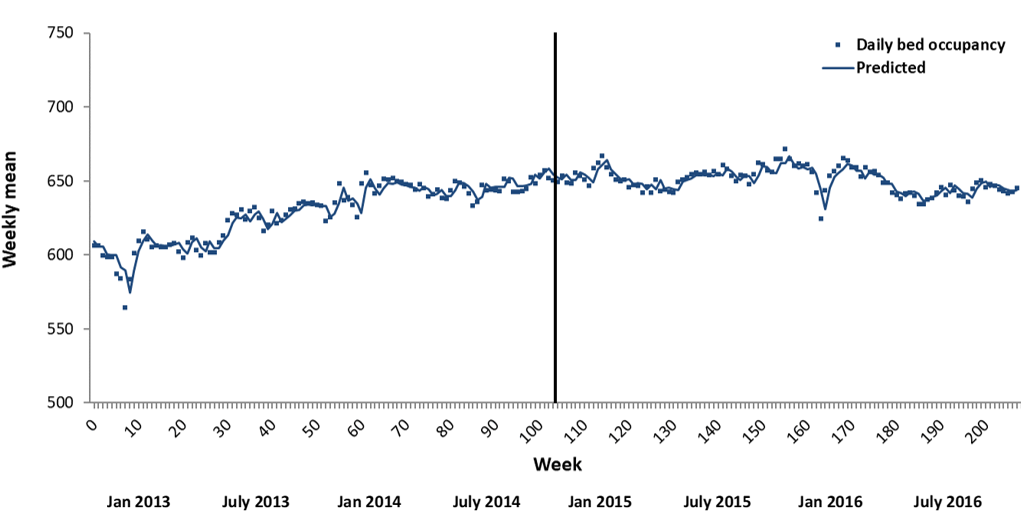
**

**Figure A.5.** The impact of PDU implementation on mean daily bed occupancy in Urban1 acute adult inpatient wards. Please note: the black vertical line represents the interruption (i.e., implementation of the PDU). A first order autoregressive term was included in the model.

**
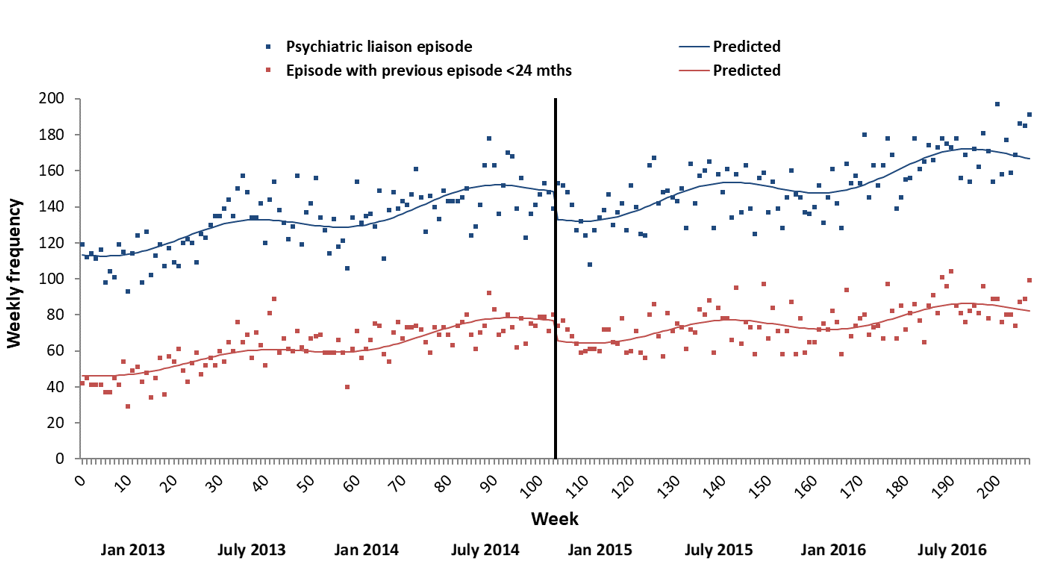
**

**Figure A.6.** The impact of PDU implementation on ED-referred psychiatric liaison episodes in Urban1. Please note: the black vertical line represents the interruption (i.e., implementation of the PDU).

**
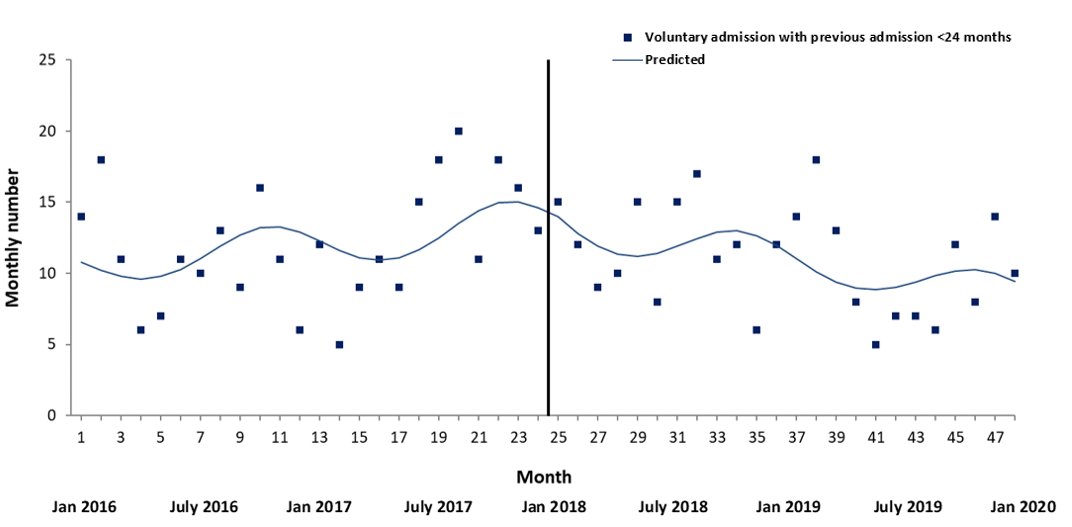
**

**Figure A.7.** The impact of PDU implementation on voluntary acute adult psychiatric inpatient admissions in Rural for service users with a previous admission in the last 24 months. Please note: the black vertical line represents the interruption (i.e., implementation of the PDU).

**
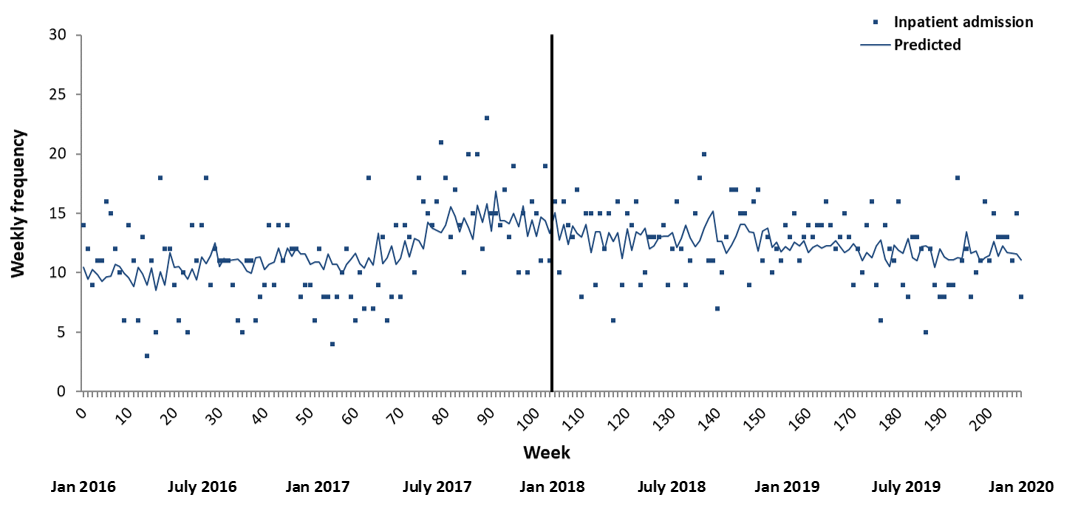
**

**Figure A.8.** The impact of PDU implementation on acute adult psychiatric inpatient admissions in Rural. Please note: the black vertical line represents the interruption (i.e., implementation of the PDU). A second order autoregressive term was included in the model.

**
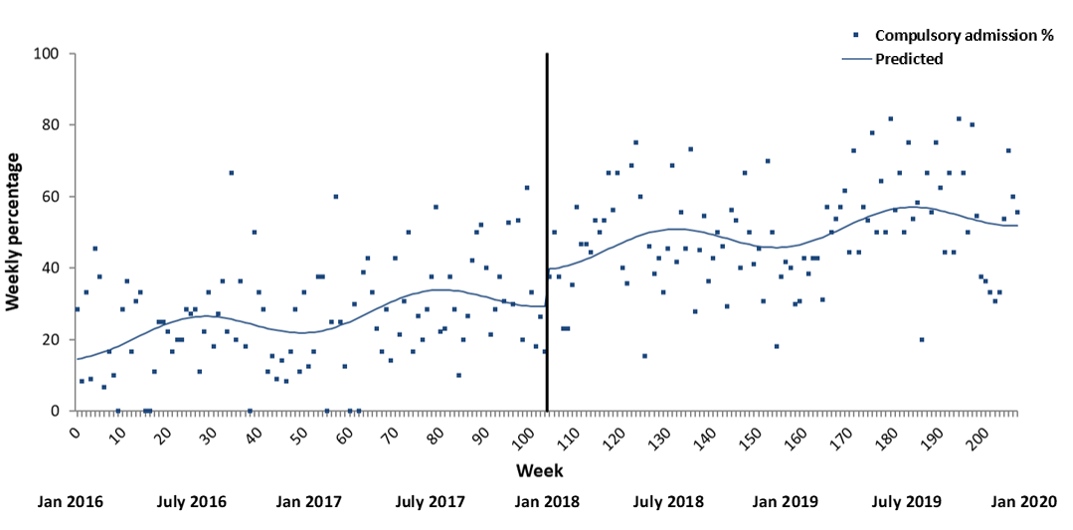
**

**Figure A.9.** The impact of PDU implementation on percentage of compulsory acute adult psychiatric inpatient admissions in Rural. Please note: the black vertical line represents the interruption (i.e., implementation of the PDU).

**
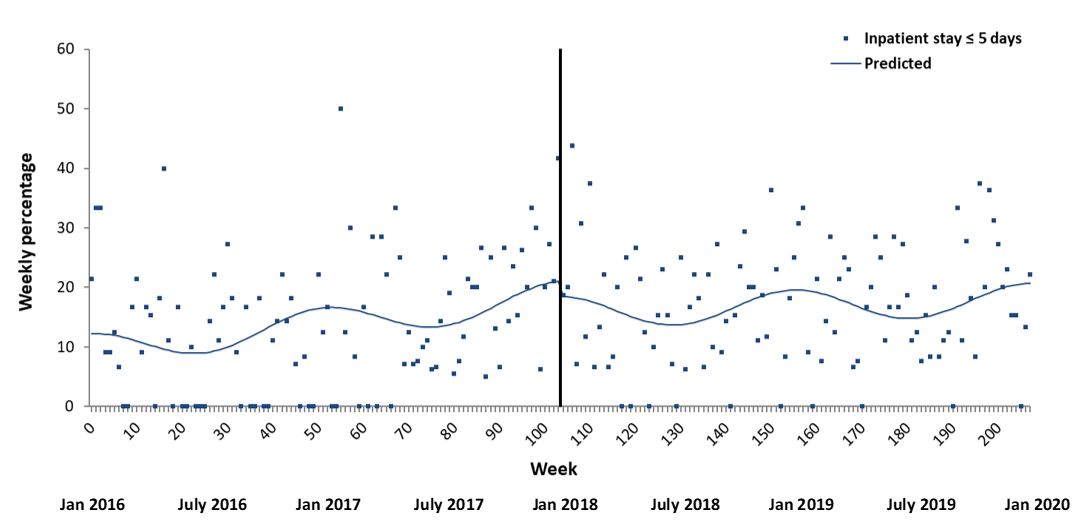
**

**Figure A.10.** The impact of PDU implementation on percentage of acute adult inpatient stays of 0-5 days in Rural. Please note: the black vertical line represents the interruption (i.e., implementation of the PDU).

**
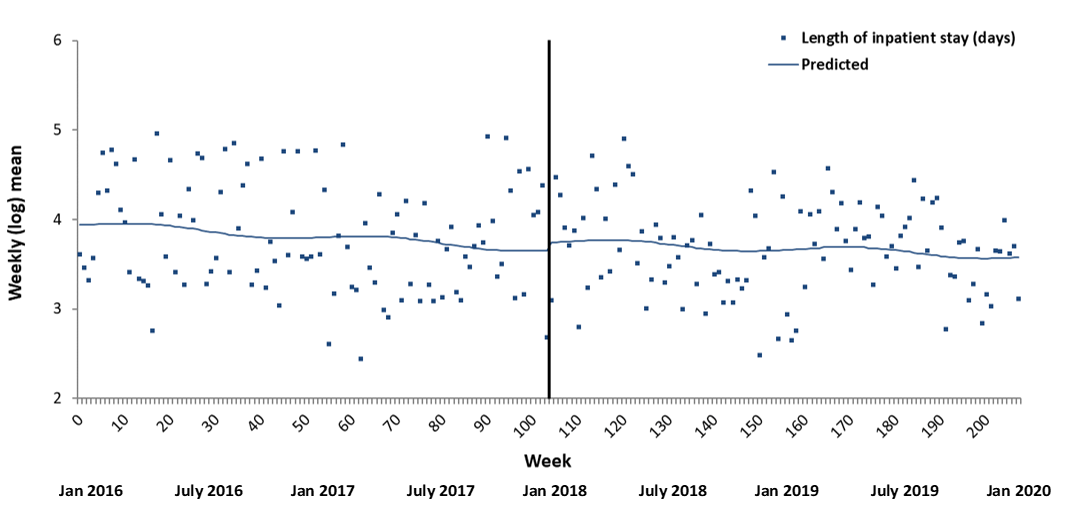
**

**Figure A.11.** The impact of PDU implementation on (log) mean length of acute adult inpatient stays in Rural. Please note: the black vertical line represents the interruption (i.e., implementation of the PDU).

**
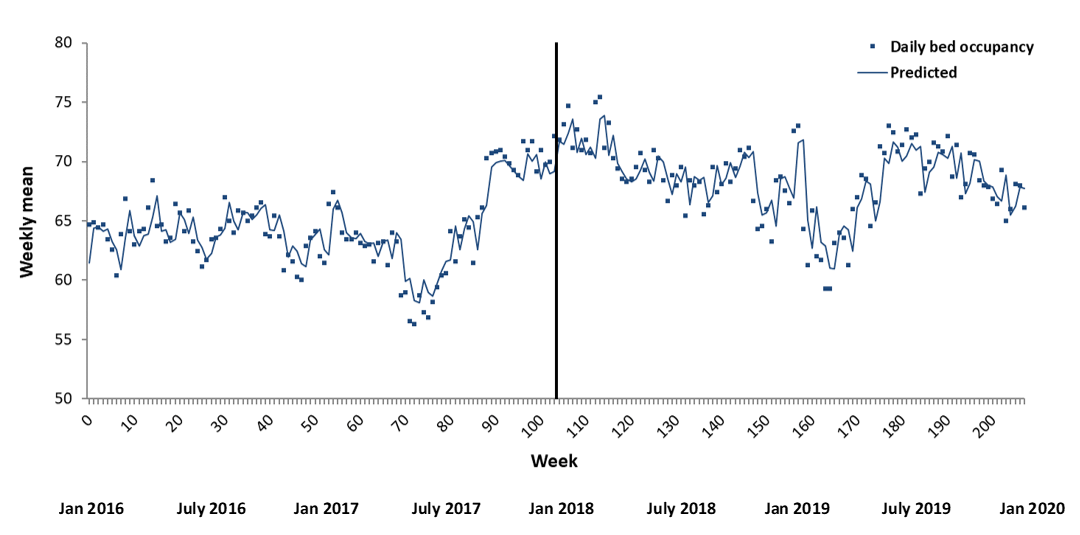
**

**Figure A.12.** The impact of PDU implementation on mean daily bed occupancy in Rural acute adult inpatient wards. Please note: the black vertical line represents the interruption (i.e., implementation of the PDU). A first order autoregressive term was included in the model.

**
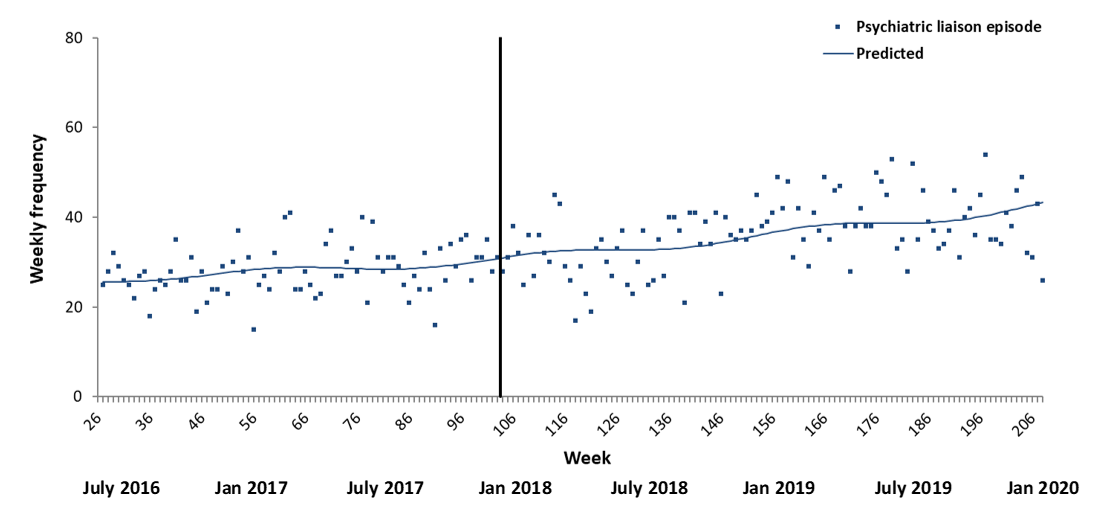
**

**Figure A.13.** The impact of PDU implementation on ED-referred psychiatric liaison episodes in Rural. Please note: the black vertical line represents the interruption (i.e., implementation of the PDU). Psychiatric liaison episode data was not available in the first 6 months of the time series in Rural.

**
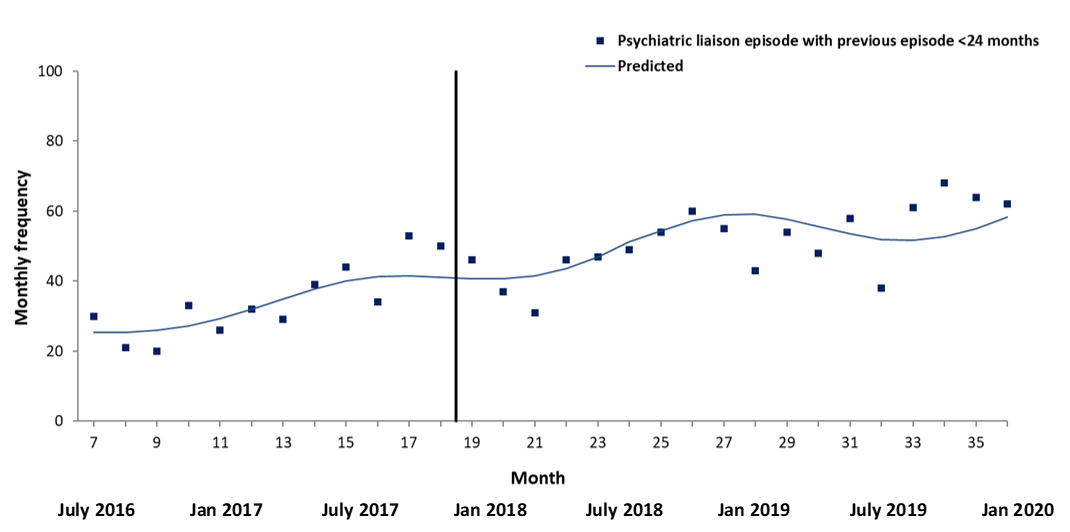
**

**Figure A.14.** The impact of PDU implementation on ED-referred psychiatric liaison episodes in Rural for service users with a previous episode in the last 24 months. Please note: the black vertical line represents the interruption (i.e., implementation of the PDU). Psychiatric liaison episode data was not available in the first 6 months of the time series in Rural.

**
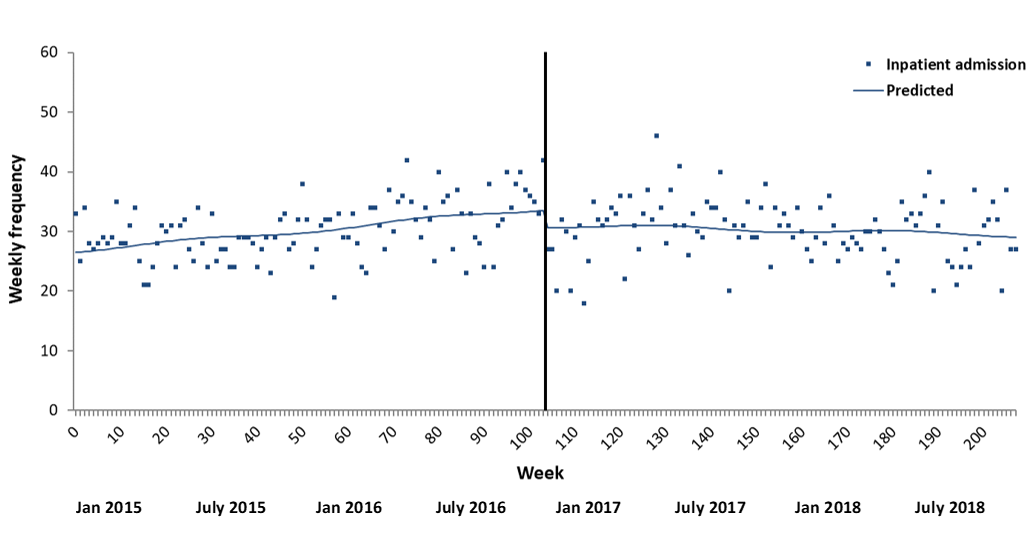
**

**Figure A.15.** The impact of PDU implementation on acute adult psychiatric inpatient admissions in Urban2. Please note: the black vertical line represents the interruption (i.e., implementation of the PDU).

**
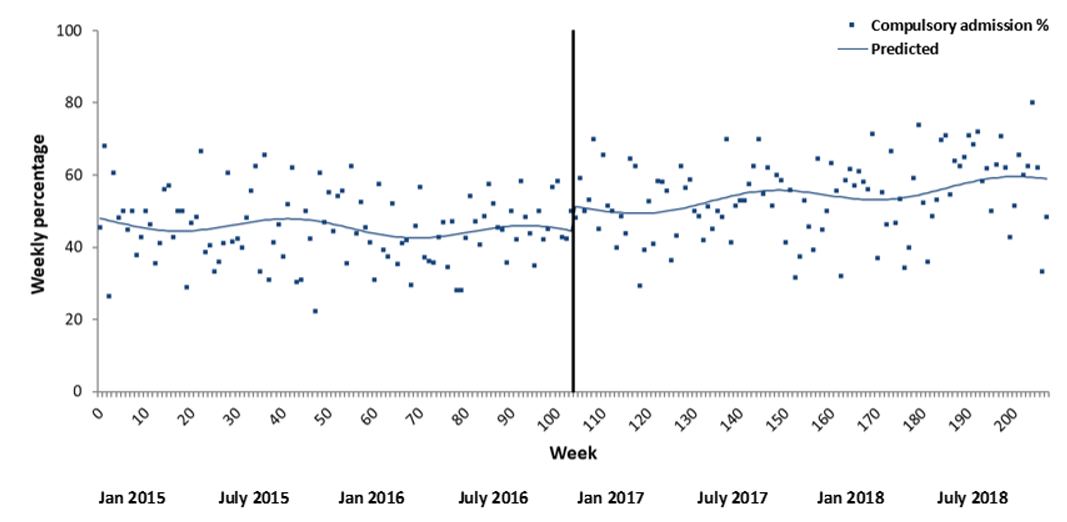
**

**Figure A.16.** The impact of PDU implementation on percentage of compulsory acute adult psychiatric inpatient admissions in Urban2. Please note: the black vertical line represents the interruption (i.e., implementation of the PDU).

**
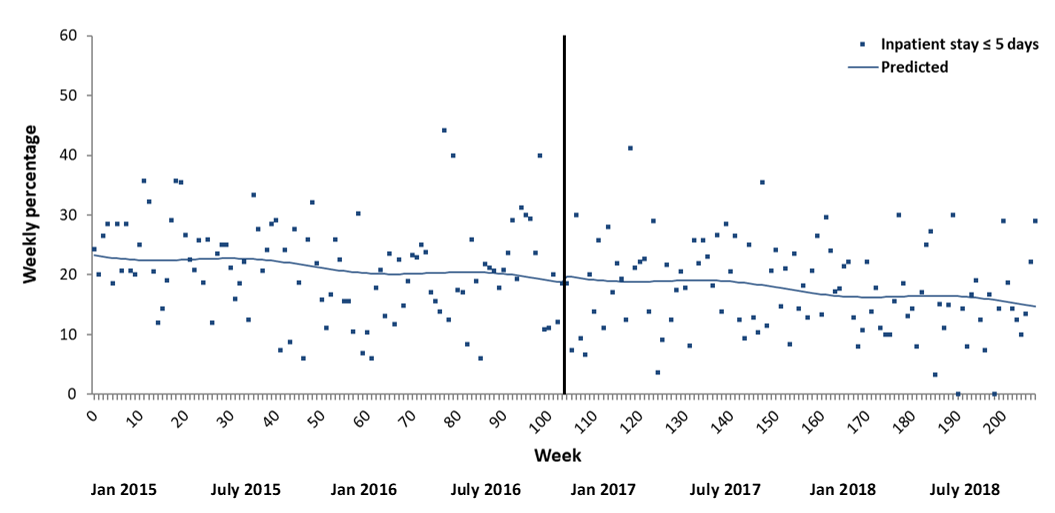
**

**Figure A.17.** The impact of PDU implementation on percentage of acute adult inpatient stays of 0-5 days in Urban2. Please note: the black vertical line represents the interruption (i.e., implementation of the PDU).

**
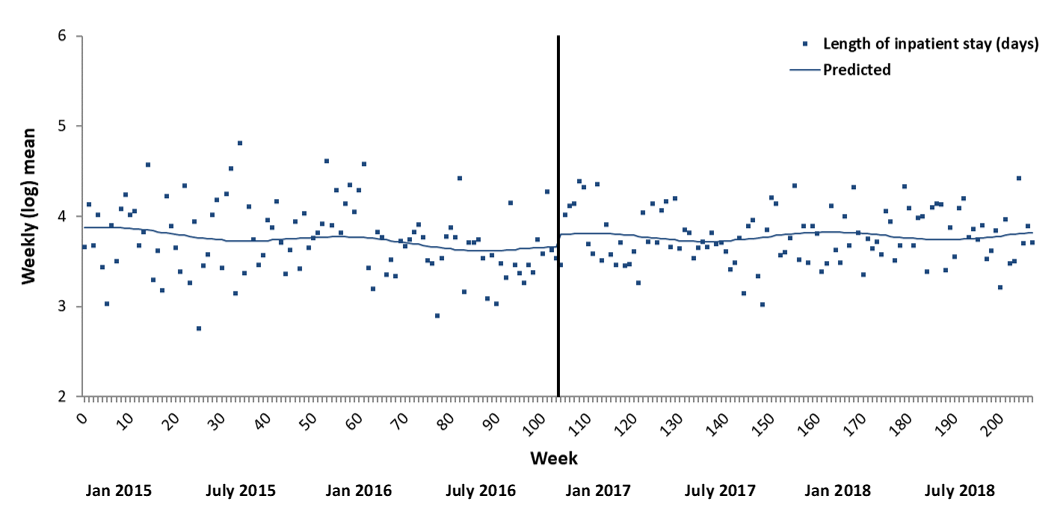
**

**Figure A.18.** The impact of PDU implementation on (log) mean length of acute adult inpatient stays in Urban2. Please note: the black vertical line represents the interruption (i.e., implementation of the PDU).

**
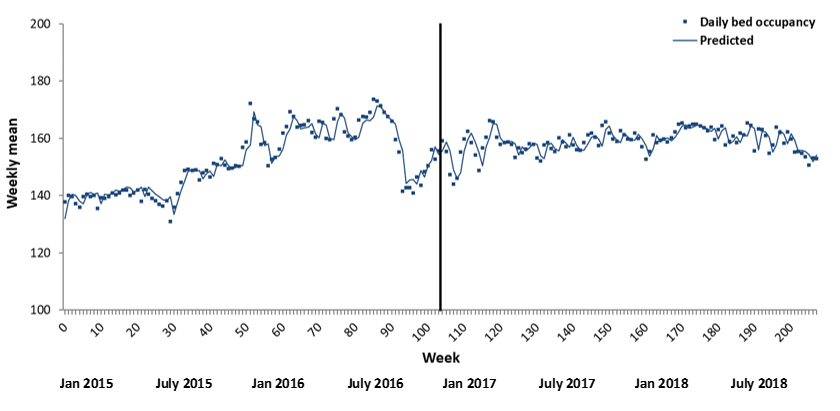
**

**Figure A.19.** The impact of PDU implementation on mean daily bed occupancy in Urban2 acute adult inpatient wards. Please note: the black vertical line represents the interruption (i.e., implementation of the PDU). A first order autoregressive term was included in the model.

**
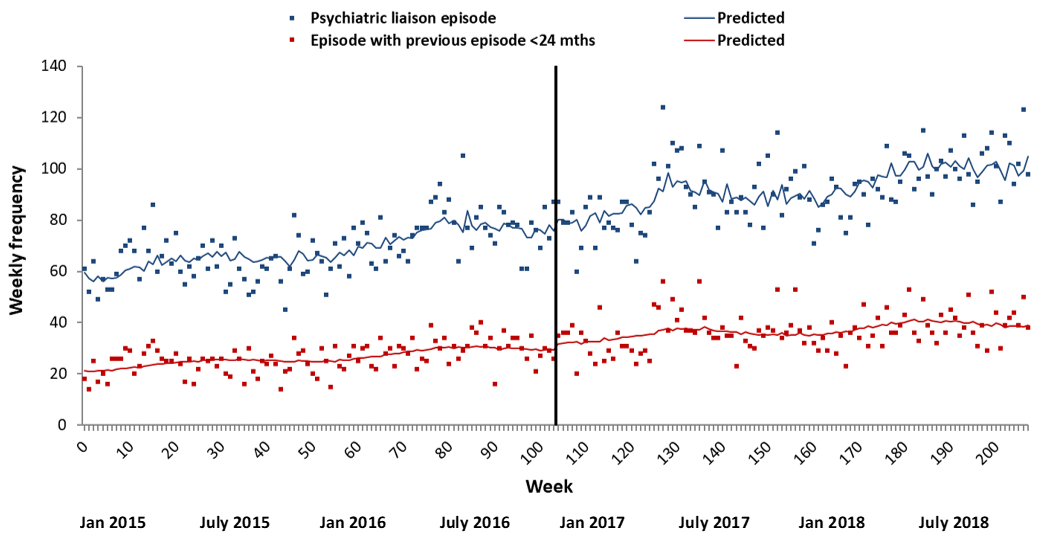
**

**Figure A.20.** The impact of PDU implementation on ED-referred psychiatric liaison episodes in Urban2. Please note: the black vertical line represents the interruption (i.e., implementation of the PDU). Autoregressive terms (first-order) were included in ED-referred psychiatric liaison episodes and ED-referred psychiatric liaison episodes with previous episode <24 months models for Urban2.

**
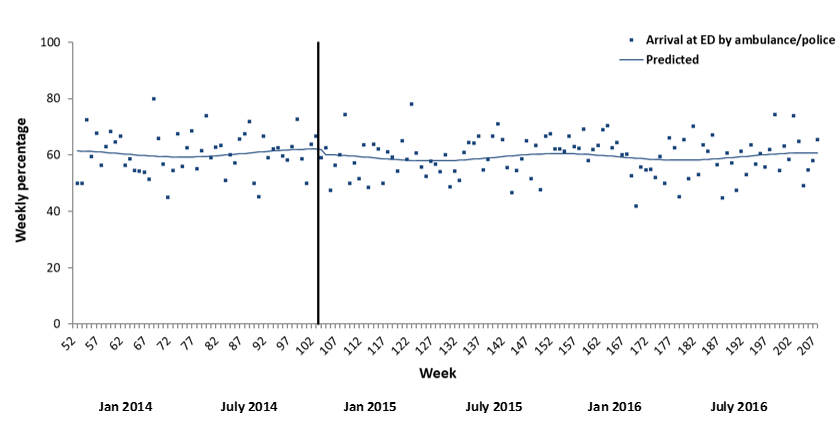
**

**Figure A.21.** Impact of PDU implementation on weekly number of mental health-related ED attendances with arrival by ambulance or police in Urban1. The black vertical line represents the interruption (i.e., implementation of the PDU). Urban1 ED attendance data was not available in the first 12 months of the time series.

**
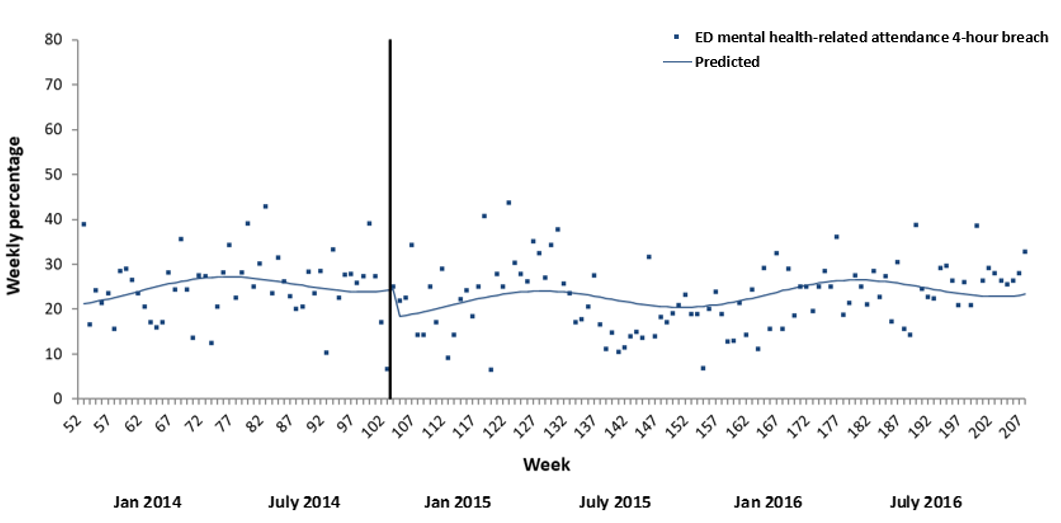
**

**Figure A.22.** Impact of PDU implementation on the proportion of mental health-related ED attendances with 4-hour breach in Urban1. The black vertical line represents the interruption (i.e., implementation of the PDU). Urban1 ED attendance data was not available in the first 12 months of the time series.

**
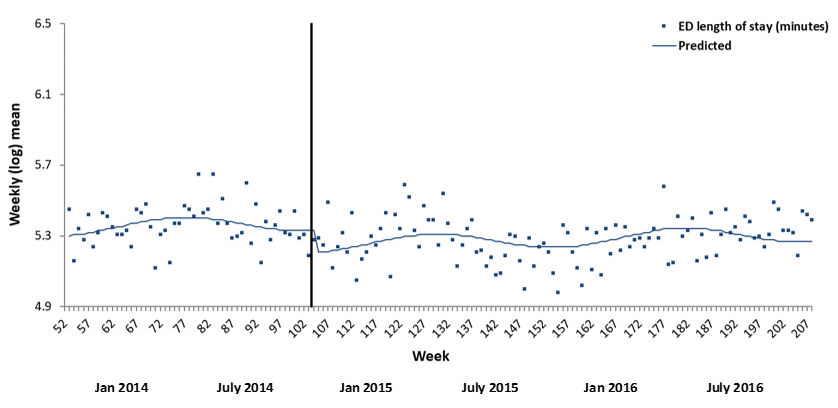
**

**Figure A.23.** Impact of PDU implementation on mean length of stay of mental health-related ED attendances in Urban1. The black vertical line represents the interruption (i.e., implementation of the PDU). Urban1 ED attendance data was not available in the first 12 months of the time series.

**
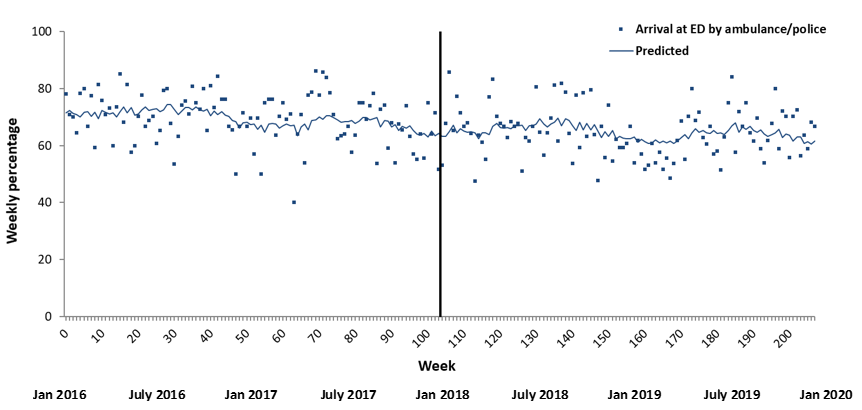
**

**Figure A.24.** Impact of PDU implementation on weekly number of mental health-related ED attendances with arrival by ambulance or police in Rural. The black vertical line represents the interruption (i.e., implementation of the PDU). A first order autoregressive term was included in the model.

**
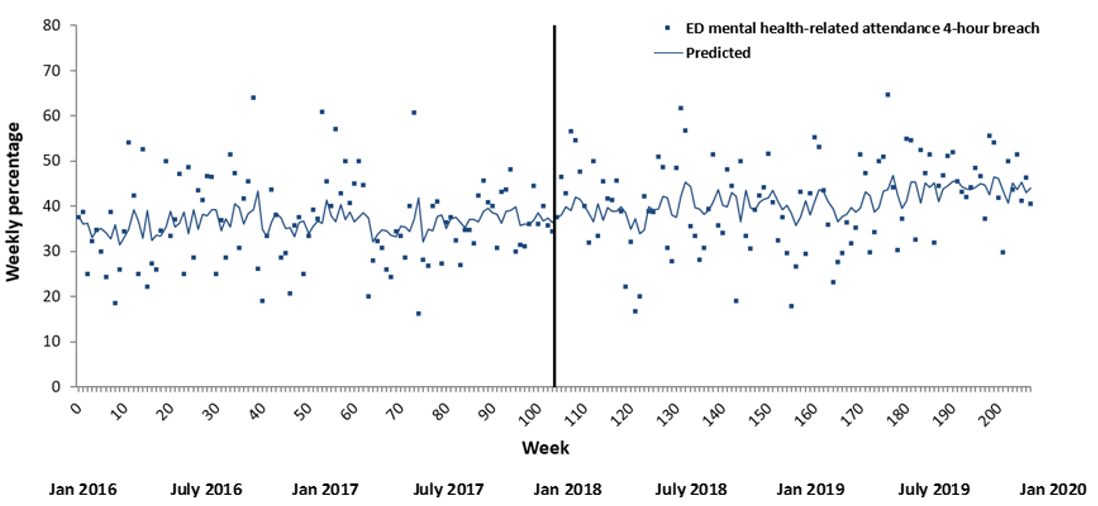
**

**Figure A.25.** Impact of PDU implementation on the proportion of mental health-related ED attendances with 4-hour breach in Rural. The black vertical line represents the interruption (i.e., implementation of the PDU). A first order autoregressive term was included in the model.

**
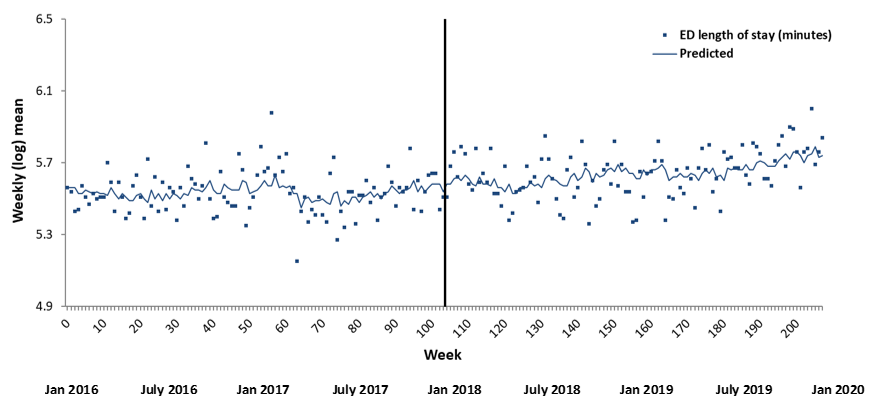
**

**Figure A.26.** Impact of PDU implementation on mean length of stay of mental health-related ED attendances in Rural. The black vertical line represents the interruption (i.e., implementation of the PDU). A first order autoregressive term was included in the model.

**
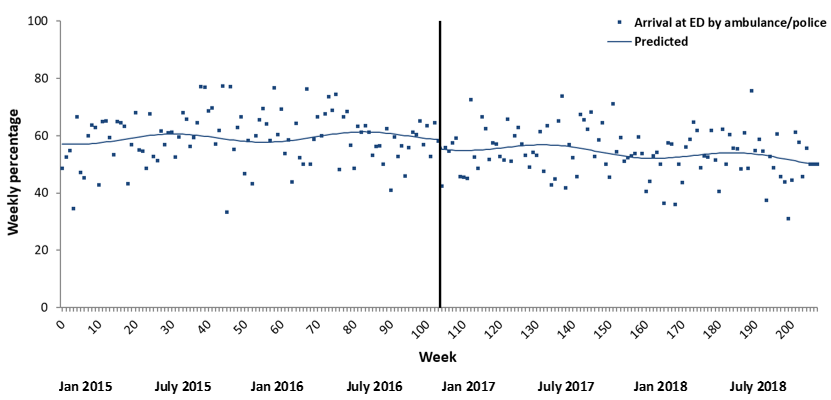
**

**Figure A.27.** Impact of PDU implementation on weekly number of mental health-related ED attendances with arrival by ambulance or police in Urban2. The black vertical line represents the interruption (i.e., implementation of the PDU).

**
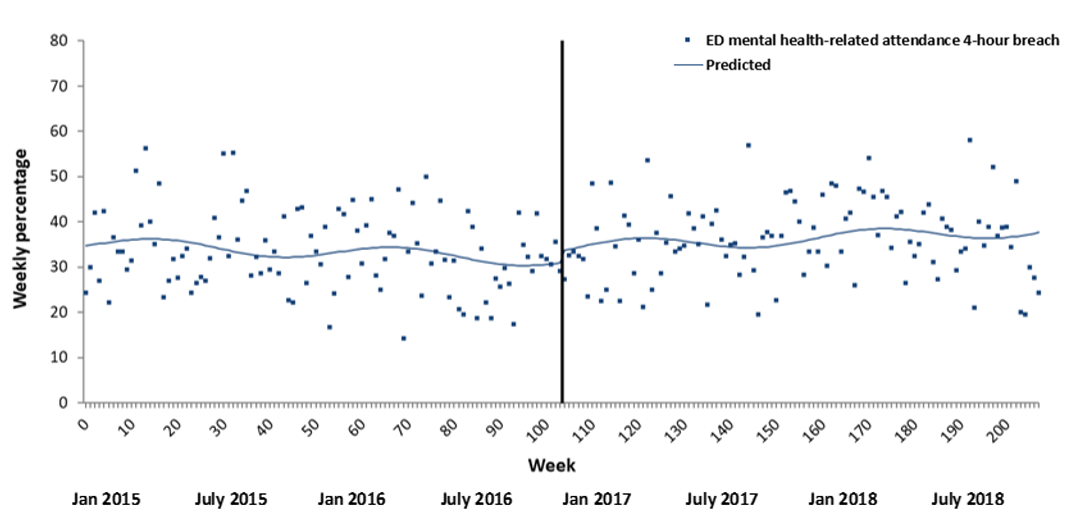
**

**Figure A.28.** Impact of PDU implementation on the proportion of mental health-related ED attendances with 4-hour breach in Urban2. The black vertical line represents the interruption (i.e., implementation of the PDU).

**
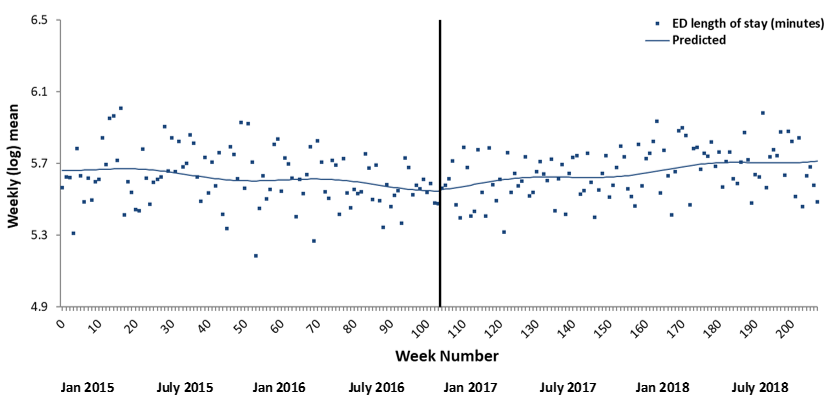
**

**Figure A.29.** Impact of PDU implementation on mean length of stay of mental health-related ED attendances in Urban2. The black vertical line represents the interruption (i.e., implementation of the PDU).
