# Supplementary material for: The effect of psychiatric decision unit services on inpatient admissions and mental health presentations in emergency departments: an interrupted time series analysis from two cities and one rural area in England
Source: Epidemiol Psychiatr Sci. 2024 Mar 21;33:e15. doi: 10.1017/S2045796024000209 (PMC11362677; doi:10.1017/S2045796024000209)
Supplement: Smith et al. supplementary material 2 — Smith et al. supplementary material [file S2045796024000209sup002.docx]

## **Supplementary appendix**

## Supplementary methods: Data collection and interrupted time series (ITS) analysis

### Data collection

The study tested the impact of a service-level change at a secular level, namely the implementation of the PDU, within the context of an ITS analysis design. Information Management Services personnel within 6 participating UK National Health Service (NHS) mental health and acute Trust hospitals provided relevant (Trust-wide) service use data. Reliable data concerning acute adult inpatient and psychiatric liaison episode activity could not be sourced from a fourth (urban) PDU site originally intended for study inclusion. Data did not include personally identifiable information and was handled in accordance with the UK Data Protection Act 2018 (incorporating the EU20 General Data Protection Regulation).

#### Psychiatric Decision Unit (PDU) data

PDU data (e.g., number of visits, length of stay, destination upon discharge) pertaining to the first 2 years of operation for each site were also collected (to provide context to any observed changes in outcomes post-PDU implementation).

#### Psychiatric hospital

Psychiatric hospital data centred on patterns of activity in acute adult inpatient wards over the relevant period, including the admission frequency and type (voluntary or compulsory), length of inpatient stay and acute adult ward bed occupancy. Inpatient admissions were classified as voluntary or compulsory according to the legal status at admission (‘informal admission’ where the service user consents to treatment and is legally allowed to leave the facility, or ‘formal detention under the Mental Health Act (MHA) for assessment and/or treatment’ where the service user may lack the capacity to consent or has refused their consent but is mandated to receive care). Recorded inpatient admissions where discharge and subsequent admission were on the same or consecutive days were considered as a single admission. Where an individual was an inpatient on an acute adult psychiatric ward at the time of data extraction, their continuous length of stay data was excluded from analyses (Rural; n=11), although categorical data concerning stay of ≤5 days/>5 days was recorded (in all of the 11 cases, stay was >5 days).

The frequency of psychiatric liaison episodes where an individual was referred from an ED was also calculated; referrals included those service users referred to Liaison Psychiatry Services irrespective of whether an assessment by Liaison Psychiatry Services was subsequently carried out. To avoid duplicate admissions for the same event, Liaison Psychiatry referrals separated by <12 hours were considered as a single episode (where subsequent referral occurred <12 hours after previous referral only the first episode of the two was considered). The number of informal (voluntary) and total acute adult admissions, acute adult ward bed occupancy and frequency of psychiatric liaison episodes were considered against the catchment population of the relevant mental health Trust in each week under study, calculated by linearly interpolating mid-year (adult) population estimates of boroughs served by the Trust, or, where appropriate, from mid-year population estimates for Clinical Commissioning Groups in England, both provided by the UK’s Office for National Statistics (ONS). ONS population estimates were available through to 2020.

#### Acute general hospital

For each (participating) acute general hospital, psychiatric presentations in the emergency department (ED) over the relevant period were extracted from ‘Presenting Complaint’/’Reason for Visit’ and ‘Diagnosis’/’ED Coding’ entries in Trust hospital ED data. Specifically, psychiatric presentations included adult attendances to a hospital ED where the presenting complaint reflected a mental or behavioural health issue and/or the primary diagnostic code was consistent with a diagnosis of either one of more mental and behavioural disorders (F01-F79 of the International Classification of Diseases, 11th edition (World Health Organization, 2011)) or self-harm (X60-X84). The specific terms used to search to initially screen for mental health attendances in ‘Presenting Complaint’/’Reason for Visit’ and ‘Diagnosis’/’ED Coding’ entries were %MENTAL%, ‘PSYC&’, ‘SUIC&’, ‘SELF&’, and ‘OVERDOSE’. Where an attending individual was recorded as presenting with alcohol intoxication without any accompanying mental health issue or diagnostic code consistent with a diagnosis of one or more mental and behavioural disorders (excluding uncomplicated alcohol abuse with intoxication), the attendance was excluded. Where the presenting complaint was listed as an ‘overdose’, in the absence of a diagnostic code consistent with a diagnosis of self-harm or one or mental and behavioural disorders, only instances where the overdose was stated as ‘deliberate’ or ‘intentional’ were included. For one participating site (Urban 1), only the presenting complaint/reason for visit was available and as such considered for including attendances.

ED hospital activity outcomes focussed on psychiatric presentation frequency, arrival method (e.g., ambulance) and length of ED stay. To avoid duplicate admissions for the same event, recorded ED visits separated by <12 hours were considered as a single episode (where subsequent visit was <12 hours after discharge only the first episode of the two was considered). The frequency of ED mental health attendances was considered relative to the corresponding hospital catchment population (HCP), calculated for each week under study by linearly interpolating (assuming a mid-year point) yearly (adult) population estimates derived for each site from the proportionate flow methods adopted by Public Health England (Brown et al., 2006, Perry, 2017). HCP information was available through to 2018; for the site where data extended beyond this year (Rural), HCPs in subsequent years were estimated via linear extrapolations for the population trend in the prior three years (2016-2018).

#### PDU and service reconfiguration/change in model of care data

PDU data (e.g., number of visits, length of stay, destination upon discharge) pertaining to the first 2 years of operation for each site was also collected (to provide context to any observed changes in outcomes post-PDU implementation). Additionally, a small number of semi-structured interviews were conducted with strategic managers in each site (e.g., PDU manager, Acute Care Pathway lead, mental health lead commissioner (or their equivalent locally), ED manager, and/or ED clinical director) to identify any changes to the crisis care pathway (e.g., introduction or withdrawal of relevant services, amendments to policy or protocol that target the assessment and/or management of psychiatric presentations in ED). Where possible, these data were accounted for in secondary analyses of primary outcome measures in additional ITS analyses with the intention of controlling for any potentially confounding changes to models of care (e.g., service reconfiguration) that occurred with the time series period of interest.

### ITS: Design and statistical analyses

#### ITS design

Changes in acute and psychiatric hospital activity following the introduction of PDUs in 3 sites were assessed via a retrospective, secular trend analysis using an interrupted time series (ITS) design considering routinely collected healthcare data. ITS are robust quasi-experimental designs that are increasingly being used to evaluate the impact of changes to health care or organisational interventions implemented in healthcare settings where randomisation of the intervention is impractical or unethical (Greenhalgh et al., 2004, Ewusie et al., 2020). Typically, they concern outcomes relevant to service delivery and/or health outcomes at a (clinical) population-level, with the intention to examine whether the data pattern observed post-intervention differs from that observed pre-intervention independent of any secular trends (Ramsay et al., 2003). The methodology of the present ITS study has previously been described (Goldsmith et al., 2020). The exposure of interest in this study was the implementation of the PDU. Acute adult psychiatric inpatient ward and mental-health related ED attendances in the 24 months prior to PDU implementation were considered unexposed, while those in the 24 months following PDU implementation were exposed.

#### ITS statistical analysis

Descriptive statistics of key service use parameters, including demographic characteristics of service users (PDUs only), were used to describe PDUs, psychiatric inpatient and ED mental-health attendance activity in participating sites. Outcome data were collated as time series over a (maximum) 48-month period for each site, aggregated to a single observation at weekly or monthly units depending on the variable under study. Segmented regression analyses were employed to evaluate whether there was a change in health care utilisation outcomes following the implementation of the PDU (Wagner et al., 2002). This method allowed the calculation of three regression coefficients that can quantify the impact of a service-level change: the underlying trend prior to PDU implementation (b1), the level change immediately following PDU implementation (b2) and the slope change from pre- to post-PDU implementation (b3).

Outcomes based on count data (e.g., psychiatric inpatient admissions, mental health-related ED attendances) followed a Poisson distribution, and segmented generalized linear models (GLM) using log-transformed rates for outcomes to stabilise variances were fit to perform the statistical analyses. The natural logarithm of population estimates for the catchment area were included in these models as an offset variable (i.e., a predictor variable with a regression coefficient fixed at 1). Where models aggregated data in weekly units, the 52^nd^, 104^th^, 156^th^ and 208^th^ weeks of the time series included an additional day (or additional two days in case of a leap year); the corresponding frequency values were adjusted to account for the additional day(s) before inclusion in models. Similarly, where models aggregated data in monthly units, frequency values were adjusted to account for the number of days in each month. Additional ITS analyses were conducted for counts of inpatient admissions, ED mental health attendances and psychiatric liaison episodes considering only those people most likely to be repeat users of these services, that is, only individuals who, in the preceding 24 months, had been admitted to psychiatric inpatient services, attended the ED (for any reason) and been referred to Liaison Psychiatry Services, respectively. Segmented linear regression (GLM with identity link) was used to evaluate outcomes based on proportions (e.g., proportion of compulsory inpatient admissions, proportion of 4-hour breaches at ED), mean length of inpatient/ED stay (log-transformed due to marked skew in data distribution) and mean daily bed occupancy.

In all segmented GLM models, robust (sandwich) variance estimators were applied to account for possible multiple admissions per patient. The presence of autocorrelation was evaluated by reviewing the (residual) autocorrelation and partial autocorrelation functions and the Durbin–Watson test statistic, and accounted for by fitting one or more lagged variables as required (first-order, second-order and/or third-order autoregressive lag variables). Since mental health care service utilisation is known to follow a seasonal pattern (Hamilton et al., 2015), terms based on (trigonometric) sine and cosine functions with a period of one year were included also (Bland, 2015, Lopez-Bernal et al., 2016). Where possible over-dispersion was identified in (log link) GLM, a scale parameter set to the model’s χ² value divided by the residual degrees of freedom was used. Residual analyses of the final models showed no significant deviations from model assumptions. Models based on weekly aggregated data were preferred in the first instance and constituted the majority of segmented regression (ITS) models in reported analyses. Refitting a sub-sample of these models (across different outcome variables) did not provide proportionately better model fits (according to Akaike’s and Schwarz’s Bayesian information criteria). In a limited number of cases, however, typically where weekly frequencies were very low, models based on monthly aggregated data were preferred.

ITS parameter estimates were presented in the form of beta values with 95% confidence intervals (95% CI) for all outcome variables, with percentage change values for count/log-transformed data (representing (predicted) percentage change in frequency/length of stay on PDU implementation (step change) or per week/month (trend change)) and percentage point change values for proportion data (representing increase/decrease in the (predicted) percentage of that variable as a result of PDU implementation (step change) or per week/month (trend change).

Subsequently, to estimate overall effects, individual site estimates of PDU implementation impact (short- and long-term) were pooled in a meta-analytical model. To minimise imprecision of pooled effect estimates, the inverse variance approach was adopted, whereby the weight assigned to each site was the inverse of the variance of the step- and trend-change estimates from fitted models (obtained by squaring the standard errors of parameter estimates) (Gebski et al., 2012). Fixed and random effect estimates were provided for all outcomes. Where the chi-squared statistic (Cochran’s Q) and associated p-value indicated heterogeneity of PDU effects across sites (variation in effect estimates beyond chance), estimates from random effects model were preferred to interpret overall effects.

Finally, secondary analyses of primary outcome measures in ITS were also performed with a view to attempt to account for the impact of any other crisis care service reconfigurations that may have been relevant to outcome measures by introducing a second break-point in the ITS models, subject to reconfigurations being sufficiently distant in time from the start/end points of the time series and the PDU implementation to distinguish any impact. Specifically, segmented regression models with multiple interventions were fitted (Linden, 2017). These utilised a similar structure to the segmented regression models described above, but also included a separate term for an additional service change (to describe any immediate effect of the change) and an additional time term to account for time since that service change (to describe a difference between the slopes of the time before and after the service change). Models were administered separately to assess the impact of each additional service change with PDU implementation.

A p-value of less than 0.05 was considered statistically significant in all analyses. Analyses were administered using Stata 16 (StataCorp., College Station, Texas) and SPSS (IBM, Version 26).

#### Sample size

Aggregating psychiatric admission and ED service use data over the relevant period (24 months pre- and post-implementation of PDUs) yielded 208 weekly or 48 monthly time points depending on (baseline) frequency of events. This is more than the 40 data points (20 pre- and 20 post-change) typically considered as adequate for valid ITS model analysis (Pawson and Tilley, 1997) and provided sufficient power to detect any existing medium effects. For instance, assuming five (or less) factors entered in a model testing a single parameter, to detect a time*slope interaction with medium effect size on an outcome, a sample of 208 time points has >99% power (calculated using G-Power, ‘linear multiple regression: fixed model, r2 increase’ module).

### Deviations from protocol

There were a small number of deviations from the methods described in the published protocol (Goldsmith et al., 2020). With respect to the plan of statistical analyses detailed in the protocol, ITS analyses concerning the weekly/monthly numbers of inpatient admissions, ED mental health attendances and psychiatric liaison episodes considered raw frequencies rather than estimates per 1000 trust catchment population. This was primarily because the observed event frequencies were typically low (e.g., <100) relative to the size of the catchment populations for mental health (700000-1000000) and acute hospital sites (340000-430000) resulting in potential rates that are difficult to interpret unless the adopted denominator is very large (e.g., 1000000) which in most cases would be more than the catchment population served by the Trust. Differences in the size of Trust catchment populations over the 4-year study period were controlled for in segmented regression models (for count data) by inclusion of the catchment population estimates across the study period as an offset variable. Although cross-site comparisons are more challenging using raw frequencies, mental health Trust sites and general hospital (acute) Trust sites shared similar catchment populations suggesting broad comparability with respect to the size of the population served by participating sites.

### References

Bland, M. 2015. *An introduction to medical statistics*, Oxford university press.

Brown, P., Ho, D., Shaw, M., Verne, J. & Trotter, C. Estimating catchment populations in the South West: why and how. UK Public Health Association Annual Conference, Telford, 2006.

Ewusie, J. E., Soobiah, C., Blondal, E., Beyene, J., Thabane, L. & Hamid, J. S. 2020. Methods, applications and challenges in the analysis of interrupted time series data: a scoping review. *Journal of Multidisciplinary Healthcare,* 13**,** 411.

Gebski, V., Ellingson, K., Edwards, J., Jernigan, J. & Kleinbaum, D. 2012. Modelling interrupted time series to evaluate prevention and control of infection in healthcare. *Epidemiology & Infection,* 140**,** 2131-2141.

Goldsmith, L. P., Smith, J. G., Clarke, G., Anderson, K., Lomani, J., Turner, K. & Gillard, S. 2020. What is the impact of psychiatric decision units on mental health crisis care pathways? Protocol for an interrupted time series analysis with a synthetic control study. *BMC Psychiatry,* 20**,** 1-9.

Greenhalgh, T., Robert, G., Macfarlane, F., Bate, P. & Kyriakidou, O. 2004. Diffusion of innovations in service organizations: systematic review and recommendations. *The Milbank Quarterly,* 82**,** 581-629.

Hamilton, I., Lloyd, C., Bland, J. M. & Savage Grainge, A. 2015. The impact of assertive outreach teams on hospital admissions for psychosis: a time series analysis. *Journal of Psychiatric and Mental Health Nursing,* 22**,** 484-490.

Linden, A. 2017. A comprehensive set of postestimation measures to enrich interrupted time-series analysis. *The Stata Journal,* 17**,** 73-88.

Lopez-Bernal, J., Cummins, S. & Gasparrini, A. 2016. Interrupted time series regression for the evaluation of public health interventions: a tutorial. *International Journal of Epidemiology,* 46**,** 348-355.

Pawson, R. & Tilley, N. 1997. *An introduction to scientific realist evaluation,* London, UK, Sage.

Perry, J. 2017. Hospital trust catchment populations: a denominator to support commissioning decisions through understanding the population likely to draw on a hospital's services. . ePoster Libr PHE. 186476.

Ramsay, C. R., Matowe, L., Grilli, R., Grimshaw, J. M. & Thomas, R. E. 2003. Interrupted time series designs in health technology assessment: lessons from two systematic reviews of behavior change strategies. *International Journal of Technology Assessment in Health Care,* 19**,** 613-623.

Wagner, A. K., Soumerai, S. B., Zhang, F. & Ross‐Degnan, D. 2002. Segmented regression analysis of interrupted time series studies in medication use research. *Journal of Clinical Pharmacy and Therapeutics,* 27**,** 299-309.

World Health Organization 2011. *ICD-11 Alpha Content Model Reference Guide, 11th Revision,* Geneva, Switzerland, World Health Organization.
